# Early Versus Delayed Anticoagulation in Acute Ischemic Stroke According to Atrial Fibrillation Subtype and Time of Diagnosis: Subgroup Analysis of the OPTIMAS Randomized Controlled Trial

**DOI:** 10.1161/STROKEAHA.125.055037

**Published:** 2026-04-01

**Authors:** James Lyon, Philip S. Nash, Norin Ahmed, Liz Aram, Maryam Balogun, Kate Bennett, Jonathan G. Best, Ekaterina Bordea, Emilia Caverly, Marisa Chau, Hannah Cohen, Mairead Cullen, Hakim-Moulay Dehbi, Caroline J. Doré, Stefan T. Engelter, Robert Fenner, Gary A. Ford, Aneet Gill, Rachael Hunter, Martin James, Archana Jayanthi, Sue Massingham, Macey L. Murray, Iwona Mazurczak, Amalia Ndoutoumou, Bo G. Norrving, Jenny Philip, Hannah Sims, Nikola Sprigg, Tishok Vanniyasingam, Nick Freemantle, Gregory Y.H. Lip, David J. Werring, on behalf of the OPTIMAS Investigators, B. Jelley

**Affiliations:** 1 Stroke Research Centre, Department of Translational Neuroscience and Stroke, University College London Queen Square Institute of Neurology, London, United Kingdom (J.L., S.N.P., L.A., J.G.B., S.M., D.J.W.).; 2 Comprehensive Clinical Trials Unit at UCL, London, United Kingdom (N.A., M.B., K.B., E.B., E.C., M. Chau, M. Cullen, H.-M.D., C.J.D., R.F., A.G., R.H., A.J., I.M., A.N., J.P., H.S., T.V., N.F.).; 3 Institute of Clinical Trials and Methodology (H.C.), University College London, United Kingdom.; 4 Research Department of Haematology (H.C.), University College London, United Kingdom.; 5 Department of Haematology (H.C.), University College London Hospitals NHS (National Health Service) Foundation Trust, United Kingdom.; 6 Comprehensive Stroke Service, National Hospital for Neurology and Neurosurgery (D.J.W.), University College London Hospitals NHS (National Health Service) Foundation Trust, United Kingdom.; 7 Neurology and Neurorehabilitation, University Department of Geriatric Medicine Felix Platter, University of Basel, Switzerland (S.T.E.).; 8 Oxford University Hospitals NHS Foundation Trust, United Kingdom (G.A.F.).; 9 Medical Sciences Division, University of Oxford, United Kingdom (G.A.F.).; 10 Royal Devon and Exeter Hospital, United Kingdom (M.J.).; 11 University of Exeter Medical School, United Kingdom (M.J.).; 12 MRC (Medical Research Council) Clinical Trials Unit at UCL, London, United Kingdom (M.L.M.).; 13 Department of Clinical Sciences and Department of Neurology, Skåne University Hospital, Lund Universityne University Hospital, Lund University, Sweden (B.G.N.).; 14 Stroke Trials Unit, Division of Mental Health and Clinical Neuroscience, Faculty of Medicine and Health Sciences, University of Nottingham, United Kingdom (N.S.).; 15 Liverpool Centre for Cardiovascular Science at University of Liverpool, Liverpool John Moores University and Liverpool Heart and Chest Hospital, United Kingdom (G.Y.H.L.).; 16 Danish Centre for Health Services Research, Department of Clinical Medicine, Aalborg University, Denmark (G.Y.H.L.).; 17 Department of Cardiology, Lipidology and Internal Medicine with Intensive Coronary Care Unit, Medical University of Bialystok, Poland (G.Y.H.L.).

**Keywords:** anticoagulants, atrial fibrillation, intracranial hemorrhage

## Abstract

**BACKGROUND:**

After acute ischemic stroke, it is uncertain whether the time of atrial fibrillation (AF) diagnosis (before or after stroke) or AF subtype (paroxysmal or permanent) modifies the treatment effect of early versus delayed direct oral anticoagulant (DOAC) initiation.

**METHODS:**

OPTIMAS (Optimal Timing of Anticoagulation After Acute Ischemic Stroke) was a randomized, parallel-group, open-label trial with blinded outcome assessment. Participants with acute ischemic stroke and AF were randomized 1:1 to early (within 4 days) or delayed (day 7–14) DOAC initiation. The primary outcome was a composite of recurrent ischemic stroke, symptomatic intracranial hemorrhage, and systemic arterial embolism. In this trial subgroup analysis, the prespecified subgroup of interest was time of AF diagnosis, classified as before or after the qualifying stroke. We also investigated AF subtype classified as persistent or paroxysmal. We fitted mixed effects logistic regression models with interaction terms between each subgroup and treatment allocation. We also investigated associations between AF time of diagnosis or subtype and outcomes using multivariable logistic regression.

**RESULTS:**

We included 3619 participants (mean age 78.0±9.9 years; 45.3% women). For AF diagnosed before stroke, 37 of 918 (4.0%) participants allocated to early DOAC had a primary outcome event versus 32 of 920 (3.5%) allocated to delayed DOAC, odds ratio, 1.17 (95% CI, 0.72–1.89), while for AF diagnosed after stroke the respective primary outcome rates were 22 of 895 (2.5%) and 27 of 886 (3.0%; odds ratio, 0.79 [95% CI, 0.45–1.40], *P*_interaction_=0.312). AF subtype did not modify the treatment effect, with odds ratios (95% CIs) for early versus late DOAC for persistent and paroxysmal AF of 1.06 (0.71–1.58) and 0.66 (0.25–1.72), respectively, *P*_interaction_=0.377. AF time of diagnosis was not associated with outcome events. Compared with paroxysmal AF, persistent AF was independently associated with an increased risk of the primary outcome (adjusted odds ratio, 2.10 [95% CI, 1.19–3.68]).

**CONCLUSIONS:**

We found no evidence that AF time of diagnosis or subtype modify the effects of early DOAC treatment. Persistent AF was independently associated with approximately double the risk of the primary outcome compared with paroxysmal AF.

**REGISTRATION:**

URL: https://www.clinicaltrials.gov; Unique identifier: NCT03759938.

Atrial fibrillation (AF) is associated with an increased risk of ischemic stroke and death.^1,2^ The risk of ischemic stroke in AF is reduced by 64%, and mortality by 26%, by long-term treatment with oral anticoagulation, when compared with control, with a reduced risk of intracranial hemorrhage (ICH) when using a direct oral anticoagulant (DOAC) in comparison to a vitamin K antagonist.^3,4^ The presentation of AF can vary from short and frequently self-limiting episodes of arrhythmia in people with otherwise normal heart rhythm (paroxysmal AF) to a continuously abnormal rhythm (persistent AF).^5,6^

It has been suggested that the timing of AF diagnosis in the context of stroke (ie, before or after stroke diagnosis) may be implicated in the risk of future vascular events.^7^

A meta-analysis published in 2022, including 21 studies and 22 566 patients diagnosed with ischemic stroke or transient ischemic attack, reported a 26% lower risk of stroke recurrence in those patients with AF diagnosed after stroke compared with those with known AF.^8^ This may, in part, be due to a lower reported incidence of vascular risk factors and structural heart disease in patients with AF diagnosed after stroke compared with known AF,^7^ leading to the hypothesis that there are distinct clinical phenotypes within the AF spectrum.^8^

Although some studies suggest that paroxysmal AF is associated with a lower incidence of ischemic stroke than persistent AF,^9–11^ others suggest no difference.^12–15^ For example, the ACTIVE-W trial (Atrial Fibrillation Clopidogrel Trial With Irbesartan for Prevention of Vascular Events) comparing oral anticoagulation to combined antiplatelet therapy with aspirin and clopidogrel (in people with AF and ≥1 risk factor for stroke) found similar annual risks of stroke or systemic embolism of 2.0 in paroxysmal AF compared with 2.2 in sustained AF,^16^ whereas an observational study in people with newly diagnosed AF found no significant association between the type of AF and risk of ischemic stroke (hazard ratio, 1.09 [95% CI, 0.95–1.25]).^17^

There are limited data in patients with acute ischemic stroke, but one observational multicenter study in 2150 patients found that those with persistent AF had a higher rate of early ischemic stroke recurrence than patients with paroxysmal AF; however, no such difference was found after adjusting for potential confounding factors, suggesting that the finding was accounted for by differences in the prevalence of relevant risk factors rather than an independent effect of AF subtype.^18^

Existing guidelines do not suggest that the timing of AF diagnosis or AF subtype should influence anticoagulation decisions, and instead focus on patient-level risk factors such as age, sex, and vascular risk factors^19,20^ to identify patients who will benefit from oral anticoagulation providing individual risk is deemed moderate or above. However, if the time of AF diagnosis or subtype independently influences stroke risk in people with acute ischemic stroke, it might potentially be relevant for decisions about anticoagulation for secondary stroke prevention, as well as providing important prognostic information.

The OPTIMAS clinical trial (Optimal Timing of Anticoagulation After Acute Ischemic Stroke) demonstrated that early initiation of DOAC within 4 days of acute ischemic stroke in patients with AF was safe and noninferior to delayed DOAC initiation for a composite outcome of ischemic stroke, ICH, unclassifiable stroke, or systemic embolism.^21^ However, it is not known whether the findings of OPTIMAS are consistent regardless of AF time of diagnosis or subtype. We, therefore, did an exploratory subgroup analysis of the OPTIMAS trial to determine: (1) whether AF time of diagnosis or subtype modified the effect of early versus delayed DOAC initiation; (2) whether AF time of diagnosis or subtype is independently associated with recurrent stroke or mortality after adjusting for potential confounding factors, including treatment allocation.

## Methods

### Data Availability Statement

Data will be made available on reasonable request from appropriately qualified researchers. Requests for access to a fully anonymized version of the study data should be directed in writing to the corresponding author and will be assessed by members of the OPTIMAS trial steering committee. A data sharing agreement will be required before any data are shared.

Details of the OPTIMAS trial have been previously published.^21,22^ Briefly, OPTIMAS was a multicenter, open-label, blinded-end point, parallel-group, phase 4, randomized controlled trial at 100 UK hospitals. To be eligible for the study, adults (ie, aged ≥18 years) presenting with a clinical diagnosis of acute ischemic stroke needed to have a history of confirmed AF. In addition, the treating physician needed to be uncertain regarding the optimal timing for DOAC initiation. Participants were randomized, stratified by stroke severity, to either early DOAC (≤4 days from stroke symptom onset, n=1814) or delayed DOAC (7–14 days from stroke symptom onset, n=1807). The trial was approved by the National Health Service Health Research Authority (South Central [Oxford B] Research Ethics Committee; reference number 19/SC/0021) and funded by the British Heart Foundation. Study sites and all investigators are listed in Table S1.

### Patient Population

Participants were recruited by appropriately trained local research team investigators if they had AF confirmed by an inpatient ECG or clearly documented in their medical records. In addition, they required a clinical diagnosis of acute ischemic stroke with symptoms lasting more than 24 hours and at least 1 form of neuroimaging (computed tomography or magnetic resonance imaging) to exclude hemorrhage or a nonstroke diagnosis.

At the time of enrollment, detailed clinical information about baseline characteristics, including vascular risk factors, medical history, and participant demographics, was collected via case report forms. For this subgroup analysis of a randomized controlled trial, data on AF time of diagnosis and subtype were collected as a data point on the structured baseline assessment form conducted at the time of trial recruitment. Data were collected as to whether AF was present historically or newly diagnosed after stroke onset. Participants were also classified as having persistent or permanent AF (including atrial flutter) or paroxysmal AF, based on all available medical records by trained stroke research practitioners.

Participants were ineligible for inclusion in the main trial if any of the after criteria were present: coagulopathy or international normalized ratio >1.7 at randomization; severe hemorrhagic transformation of the index infarct (ECASS [European-Australasian Acute Stroke Study] PH2 [Parenchymal Hematoma 2])^23^ or acute ICH unrelated to infarct; contraindication to DOAC use (eg, severe renal failure with creatinine clearance <15 mL/min, or cirrhosis with Child Pugh criteria B or C); definite indication for vitamin K antagonist use; or pregnancy or breastfeeding. Participants were also ineligible for enrollment if their AF was secondary to mitral stenosis. Written informed consent was provided by all participants or an appropriate consultee according to national regulations.

### Randomization and Treatment

Participants were randomly assigned in a 1:1 ratio to early DOAC initiation (within 4 days of stroke onset) or delayed DOAC initiation (7–14 days after onset) using an independent online randomization service with random permuted blocks and randomly varying block lengths. This was stratified by stroke severity, as determined by the clinical National Institutes of Health Stroke Scale (NIHSS) score at the time of randomization (ie, 0–4, 5–10, 11–15, 16–21, or >21).

Once randomized to either the early or delayed DOAC group, the exact timing of DOAC initiation within each specified timeframe was at the treating physician’s discretion. Any DOAC currently licensed for stroke prevention in AF could be used (ie, apixaban, dabigatran, rivaroxaban, and edoxaban) with the dose and route of administration in accordance with standard clinical care and existing guidance.^24–27^

### Outcomes

The primary outcome for the OPTIMAS trial was a composite of recurrent ischemic stroke, symptomatic ICH (including hemorrhagic transformation of the qualifying infarct), unclassifiable stroke (ie, a clinical stroke syndrome without available neuroimaging for clinical reasons), or systemic embolism at 90 days. Data were collected by trained local investigators after randomization in a modified intention-to-treat population. Although blinding of treating clinicians was not possible, an independent external committee of expert clinicians, masked to treatment allocation, adjudicated all reported primary outcome events. Secondary outcomes reported in this analysis include recurrent ischemic stroke, systemic embolism, symptomatic ICH, and all-cause mortality at 90 days.

AF time of diagnosis (ie, known before or after qualifying ischemic stroke) was a prespecified subgroup analysis in the OPTIMAS statistical analysis plan,^28^ so was the primary exposure of interest. To explore aspects of AF further, we also performed a post hoc subgroup analysis according to persistent (including atrial flutter) versus paroxysmal AF. We also performed post hoc analyses of the prognostic associations of AF time of diagnosis and AF subtype to predict recurrent stroke and death. As such, these analyses should be regarded as hypothesis-generating.

### Statistical Analysis

In the OPTIMAS trial, analyses followed the modified intention-to-treat principle, meaning participants inadvertently enrolled without confirmed AF and ischemic stroke were excluded.

Numerical data were described using mean (SD) and median (interquartile range) depending on variable distributions. Categorical variables were described using number (%).

To determine whether AF time of diagnosis or subtype modifies the effect of early DOAC initiation, we fitted mixed effects logistic regression models with interaction terms separately between AF time of diagnosis and treatment allocation and AF subtype and treatment allocation. We adjusted for clustering within study centers by including random intercept terms for site in the models. The stratifying variable, NIHSS score at randomization, was included in the models as in the main OPTIMAS trial. We illustrated our results using Forest plots.

To investigate whether AF time of diagnosis or subtype was independently associated with outcome events, we used the widely adopted approach recommended by Harrell et al for developing prognostic models.^29^ We fitted univariable and multivariable mixed effects logistic models with each outcome of interest as the dependent variable. We chose prespecified independent variables based on existing evidence and biological plausibility, including age, sex, treatment group, randomization NIHSS score category, hypertension, diabetes, hypercholesterolemia, ischemic heart disease, heart failure, any previous stroke, current smoking, and thrombolysis treatment. For each outcome we fitted 2 multivariable models: model 1 included all prespecified covariates; in model 2 we assessed the stability of the estimates for the independent variable of interest by performing backwards elimination sequentially excluding variables for which *P*≥0.1 and retaining all variables with *P*<0.1 in the final model, as recommended by Harrell et al.^29^ Age, sex, DOAC treatment allocation and randomization NIHSS score category were forced into the final models regardless of *P* value. To illustrate our results, Kaplan-Meier curves were generated for each outcome according to AF time of diagnosis and AF subtype with accompanying multivariable Cox hazard ratios, including the same covariates as model 1 above.

All analyses were performed using STATA 18 (version 4.4.1) by J.L. and P.S.N.

## Results

### Baseline Characteristics

**Figure 1.**
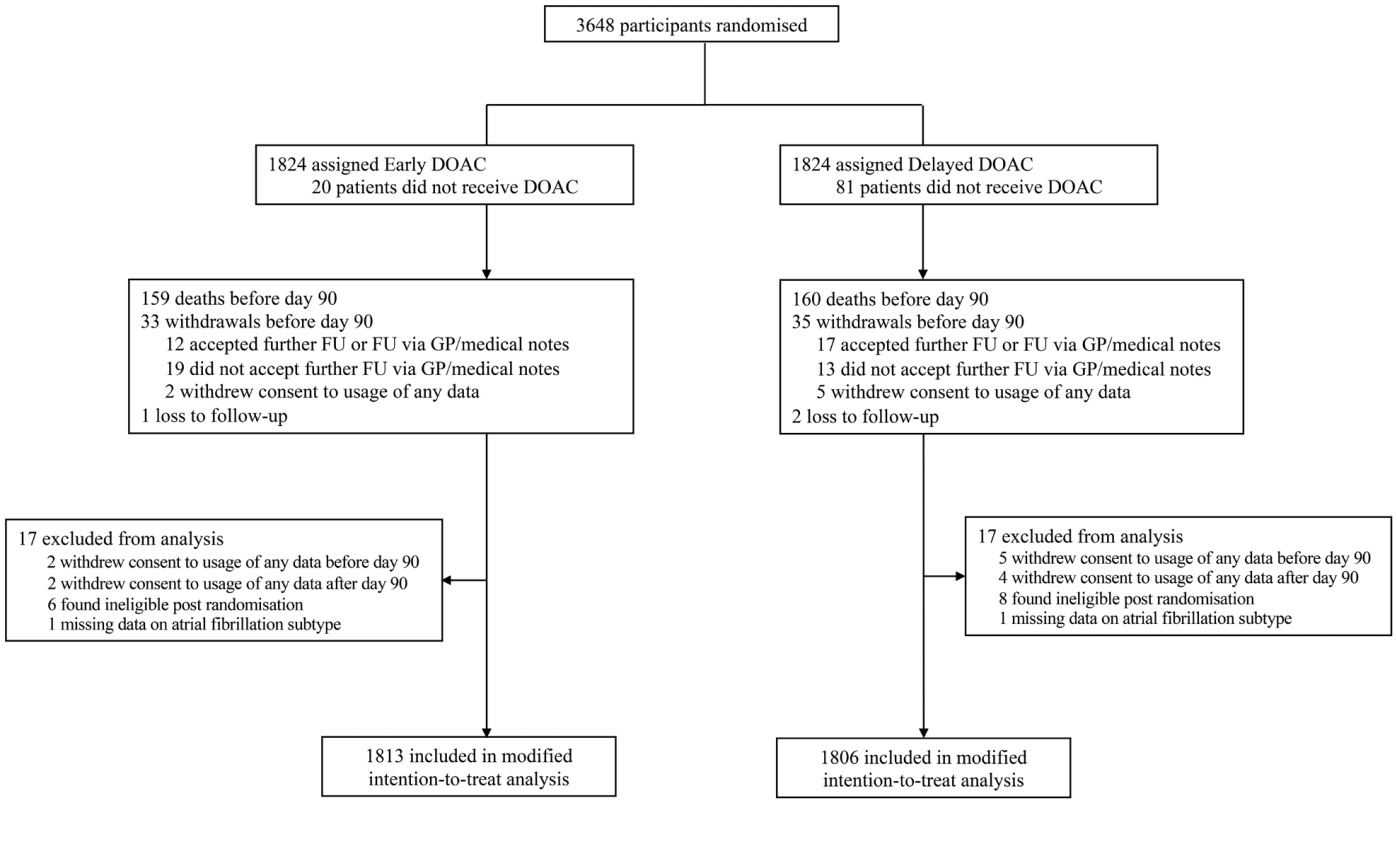
**CONSORT diagram (Consolidated Standards of Reporting Trials) of participant flow.** DOAC indicates direct oral anticoagulant; FU, follow-up; and GP, general practitioner.

Of the 3621 participants included in the OPTIMAS trial, data were available on AF time of diagnosis and subtype in 3619 participants (mean age, 78 SD [9.9]; 45.3% women). Participant flow through the trial is shown in Figure 1. Table 1 shows the baseline characteristic of participants according to AF time of diagnosis.

**Table 1.**
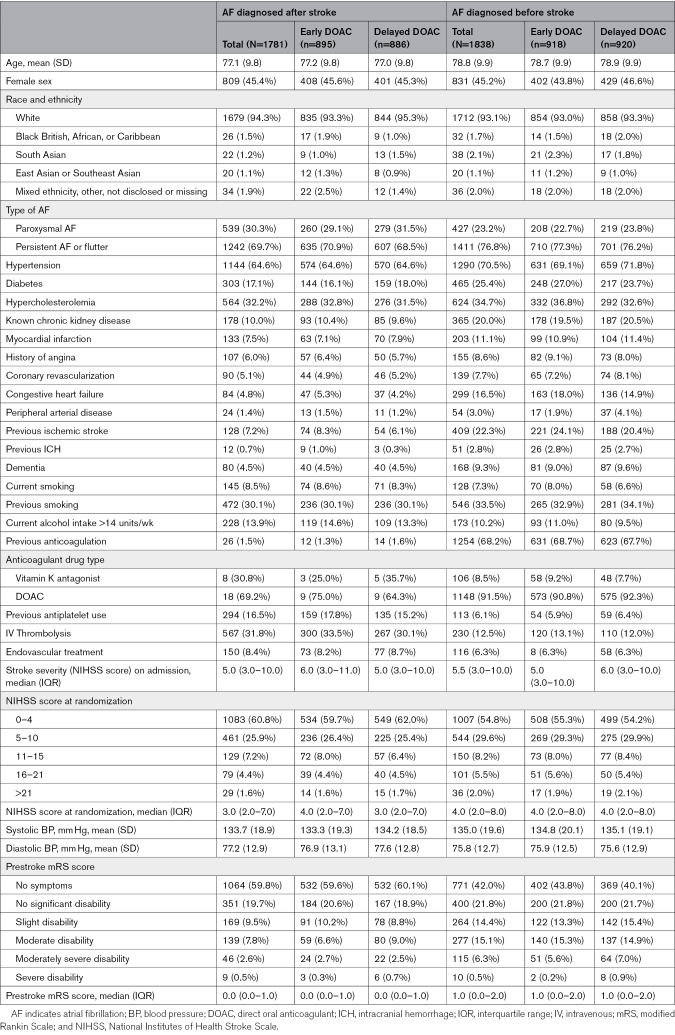
Baseline Characteristics According to AF Time of Diagnosis and Treatment Allocation

Compared with participants who had AF diagnosed after stroke, participants with preexisting AF were older (mean age 78.8±9.9 versus 77.1±9.8 years) with a higher prevalence of hypertension (70.5% versus 64.6%), higher index stroke severity (median [interquartile range] NIHSS score at randomization 4.0 [2.0–8.0] versus 3.0 [2.0–7.0]) and higher levels of disability at baseline with median (interquartile range) modified Rankin Scale score of 1 (0–2) versus 1 (0–1). Persistent AF was more prevalent in both groups compared with paroxysmal AF, although there was a higher prevalence of paroxysmal AF in newly diagnosed AF compared with preexisting AF (30.3% versus 23.2%). Overall, 68.2% of those with preexisting AF were taking anticoagulation before index stroke compared with 1.5% of participants with AF diagnosed after stroke. Conversely, 16.5% of participants with AF diagnosed after stroke were taking antiplatelets before admission compared with 6.1% in those with preexisting AF. There were substantial differences between groups in terms of reperfusion therapy, with a higher prevalence of both thrombolysis (31.8% versus 12.5%) and endovascular treatment (8.4% versus 6.3%) in those with AF diagnosed after stroke compared with AF diagnosed before stroke.

Within each study group (AF diagnosed before or after stroke), participant characteristics were well balanced between those receiving early and delayed DOAC (Table 1).

The baseline characteristic according to AF subtype is provided in Table S2.

Compared with participants who had paroxysmal AF, participants with persistent AF were older (mean age, 78.5 versus 76.4), with a higher prevalence of hypertension (68.5% versus 63.8%), and higher index stroke severity (median NIHSS score at randomization, 4.0 versus 3.0).

### AF Time of Diagnosis as an Effect Modifier for DOAC Timing

There was no significant difference in the primary outcome event rate in participants diagnosed with AF before or after stroke according to treatment allocation, and no significant treatment interaction (Table 2). In newly diagnosed participants, the primary outcome occurred in 22 of 895 (2.5%) participants receiving early DOAC versus 27 of 886 (3.0%) in those receiving delayed DOAC (odds ratio [OR], 0.79 [95% CI, 0.45–1.40]). In those with an AF diagnosis before stroke, the primary outcome occurred in 37 of 918 (4.0%) of participants in the early DOAC group versus 32 of 920 (3.5%) in the delayed group (OR, 1.17 [95% CI, 0.72–1.89]; *P*_interaction_=0.312). The rate of symptomatic ICH events was balanced between treatment allocation, and there was no significant treatment interaction with time of AF diagnosis (*P*_interaction_=0.937; Table 2).

**Table 2.**
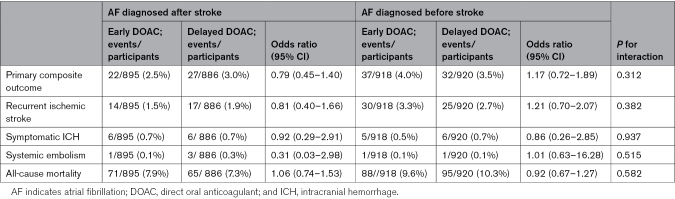
Outcome Events and Subgroup Analyses According to AF Time of Diagnosis and Treatment Allocation

### AF Subtype as Effect Modifier for DOAC Timing

There was no significant difference in outcome event rates in participants with paroxysmal AF or persistent AF according to treatment allocation, with no significant treatment interaction (Table 3). In those with paroxysmal AF the primary outcome occurred in 7 of 468 (1.5%) of participants allocated to early DOAC versus 11 of 498 (2.2%) of participants allocated to delayed DOAC (OR, 0.66 [95%, CI 0.25–1.72]), whereas in those with persistent AF the primary outcome occurred in 52 of 1345 (3.9%) participants allocated to early DOAC versus 48 of 1308 (3.7%) allocated to delayed DOAC (OR, 1.06 [95% CI, 0.71–1.58]; *P*_interaction_=0.377). The rate of symptomatic ICH events was balanced between treatment allocation, and there was no significant treatment interaction (*P*_interaction_=0.951; Table 3).

**Figure 2.**
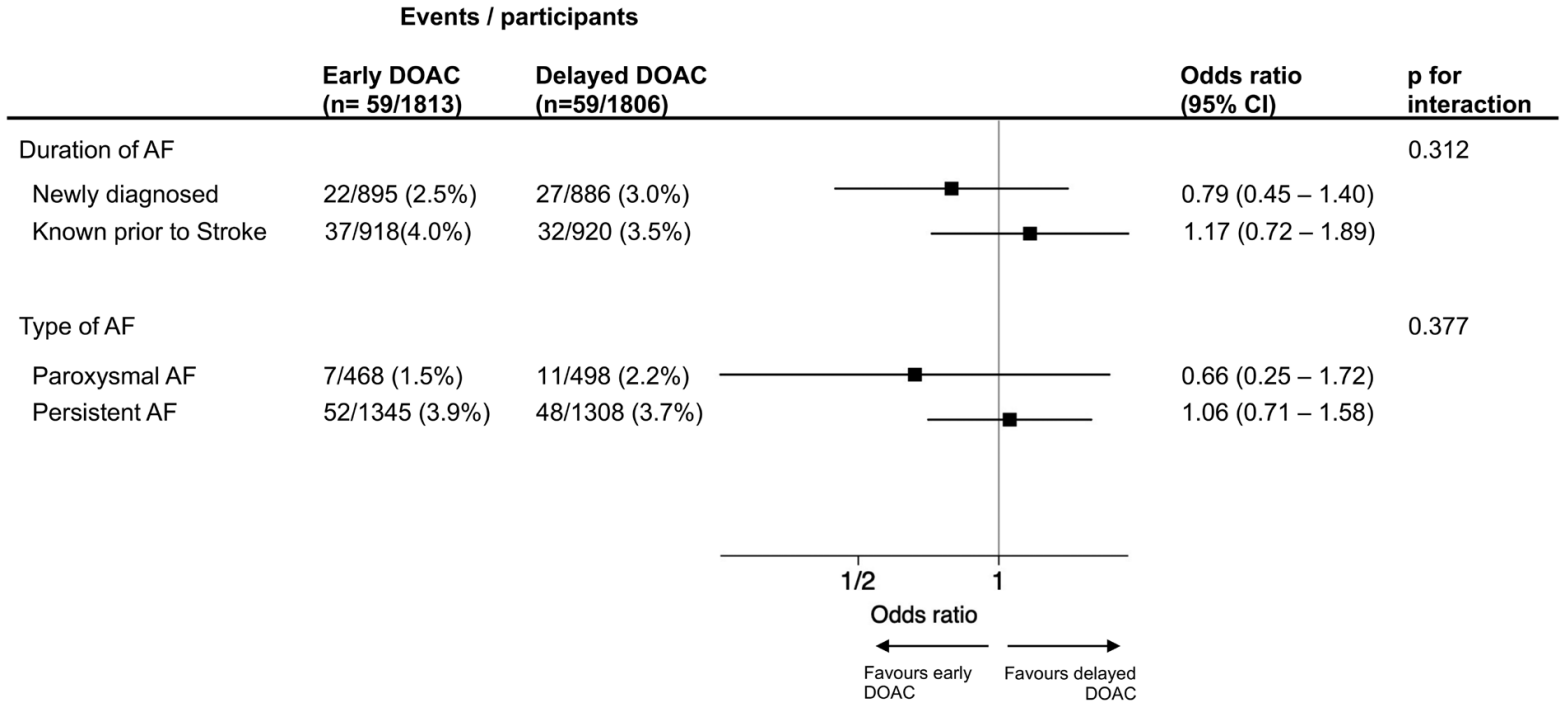
**Forest plot showing primary composite outcome events according to atrial fibrillation (AF) time of diagnosis, AF subtype, and treatment allocation.** DOAC indicates direct oral anticoagulant.

Figure 2 shows the Forest plot for the primary composite outcome according to treatment allocation, AF time of diagnosis (before or after stroke), and AF subtype (persistent versus paroxysmal).

### Outcome Events According to Timing of AF Diagnosis (Before or After Stroke)

There were 49 primary outcome events (2.8%) in those with AF diagnosed after stroke, and 69 events (3.8%) in those with known AF. The primary outcome was not associated with AF time of diagnosis in multivariable mixed effects logistic regression, adjusted OR, 1.03 (95% CI, 0.68–1.58); nor was it associated with any outcome. The adjusted ORs (95% CIs) for AF known before stroke compared with newly diagnosed were 1.23 (0.74–2.04), 0.84 (0.34–2.06), and 1.01 (0.76–1.35) for recurrent ischemic stroke, symptomatic ICH, and 90-day mortality, respectively (Table 4).

**Table 3.**
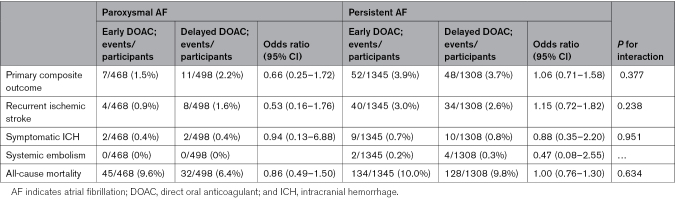
Outcome Events and Subgroup Analyses According to AF Subtype and Treatment Allocation

**Table 4.**
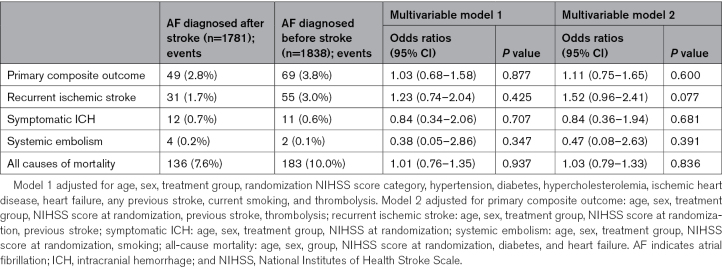
Multivariable Mixed Effects Logistic Regression Models for Outcome Events According to AF Time of Diagnosis

### Outcome Events According to Persistent or Paroxysmal AF Subtype

There were 18 primary outcome events in the group with paroxysmal AF (1.9%) and 100 events in those with persistent AF (3.8%), and compared with paroxysmal AF there was an independent association of the primary outcome with persistent AF, adjusted OR, 2.10 (95% CI, 1.19–3.68) in model 1 and 1.96 (95% CI, 1.17–3.27) in model 2 (Table 5). There were also independent associations of persistent AF with recurrent ischemic stroke in both models (adjusted OR, 2.63 [95% CI, 1.30–5.35] in model 1). All-cause mortality was not associated with AF subtype. All outcome events are shown in Table 5, and full models for the primary outcome according to time of AF diagnosis and AF subtype are shown in Table S3 and Table 6, respectively.

**Table 5.**
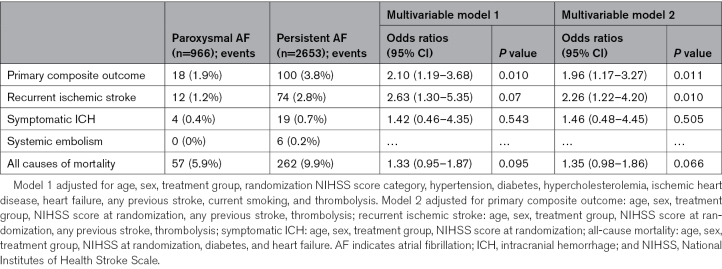
Multivariable Mixed Effects Logistic Regression Models for Outcome Events According to AF Subtype

**Table 6.**
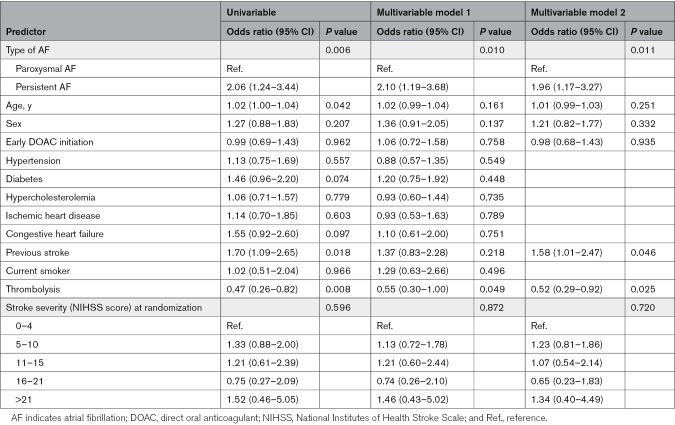
Univariable and Multivariable Mixed Effects Logistic Regression Models Showing Predictors of the Composite Primary Outcome According to AF Subtype

**Figure 3.**
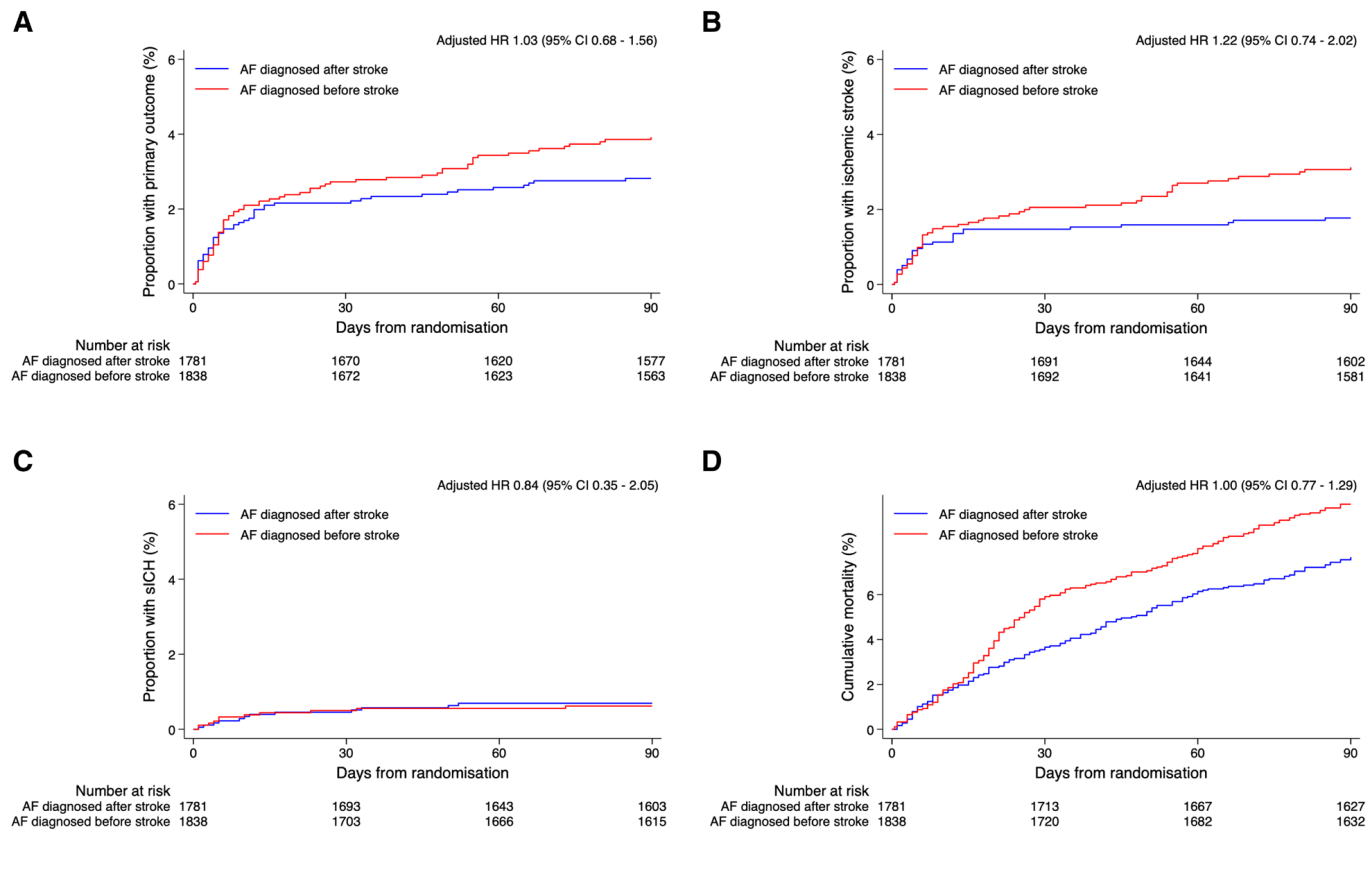
**Kaplan-Meier curves comparing outcome events according to atrial fibrillation (AF) time of diagnosis. A**, Primary outcome. **B**, Recurrent ischemic stroke. **C**, Symptomatic intracranial hemorrhage (sICH). **D**, All-cause mortality at 90 days.

**Figure 4.**
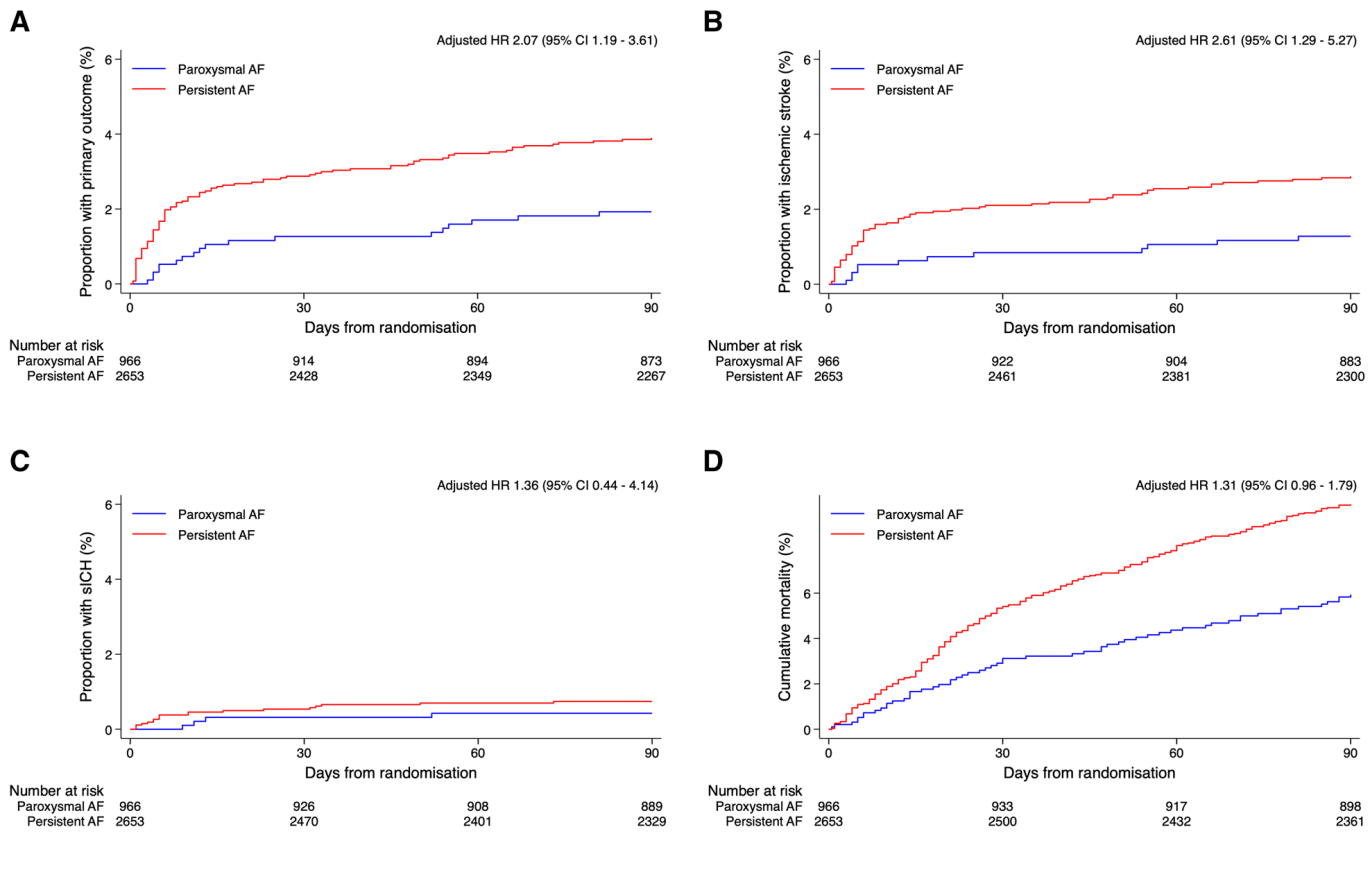
**Kaplan-Meier curves comparing outcome events at 90 days according to atrial fibrillation (AF) subtype. A**, Primary outcome, (**B**) recurrent ischemic stroke, (**C**) symptomatic intracranial hemorrhage (sICH), (**D**) all-cause mortality.

Figures 3 and 4 show the Kaplan-Meier curves and accompanying adjusted hazard ratios for each outcome event according to AF time of diagnosis and AF subtype, respectively.

## Discussion

This subgroup analysis of the OPTIMAS trial provides several important observations. First, we found no evidence that AF time of diagnosis or subtype modifies the effect of early versus delayed DOAC initiation after acute ischemic stroke. Clinicians can, therefore, be reassured that regardless of AF time of diagnosis or subtype, initiation of early DOAC is safe and noninferior to delayed DOAC initiation as demonstrated in the main OPTIMAS trial findings.^22^ This might inform future national and international guidelines. Secondly, we found an independent association of persistent AF (by comparison with paroxysmal AF) with risk of future ischemic stroke when adjusted for prognostically important potential confounding factors, and comparable risk of symptomatic ICH between groups. This could inform clinicians on anticoagulant decisions for patients at high risk of both ischemic stroke and ICH.

Our findings add to growing evidence suggesting that AF subtype (persistent versus paroxysmal) is an independent risk factor for recurrent ischemic stroke. Although several previous studies reported a higher ischemic stroke risk in patients with persistent compared with paroxysmal AF,^9,30–32^ most of the participants included did not have a previous stroke. A systematic review and meta-analysis of subgroup analysis from 6 randomized controlled trials comparing the efficacy and safety of oral anticoagulation or antiplatelet therapy in 69 990 nonvalvular AF patients reported a lower risk of ischemic stroke (risk ratio, 0.72 [95% CI, 0.59–0.87]) in paroxysmal AF.^30^ Our data expand on these observations by reporting a higher rate of early recurrence in patients with persistent AF and recent ischemic stroke, followed up for 90 days.

Our observations of a higher stroke risk in patients with persistent AF are not reflected in current guidelines,^20^ which emphasize AF risk stratification models such as CHA_2_DS_2_VASc.^33^ By contrast, our findings suggest that AF subtype might be relevant for clinical decisions in stroke secondary prevention. For example, the increased stroke risk we observed in persistent AF might justify more intensive prevention treatment strategies. A recent observational study of populations with “breakthrough stroke” (ie, ischemic stroke and AF despite anticoagulation) showed a two-thirds lower risk of stroke recurrence in those treated with left atrial appendage occlusion.^34^ While this study is subject to possible bias and residual confounding, a randomized trial is currently recruiting, which should help to answer this question (ELAPSE [Early Closure of Left Atrial Appendage for Patients With Atrial Fibrillation and Ischemic Stroke Despite Anticoagulation Therapy], https://www.clinicaltrials.gov; Unique identifier: NCT05976685). Subgroup analysis of ELAPSE could assess whether those with persistent AF benefit more from left atrial appendage occlusion intervention, and future trials could investigate persistent AF treated with left atrial appendage occlusion regardless of breakthrough stroke.

The underlying mechanisms for an increased stroke risk in persistent AF remain unclear. The continuous presence of AF leads to persistent atrial remodeling, including structural changes and fibrosis, which can promote stasis of blood flow and increase the likelihood of thrombus formation.^35^ In addition, in comparison with paroxysmal AF, persistent AF is frequently associated with greater left atrial enlargement, reducing effective contraction and further contributing to blood stasis and thrombo-embolism.^36^ The prolonged exposure to irregular heart rhythms may also impair endothelial function.^35^ Patients with persistent AF also have higher markers of platelet activation compared with those in paroxysmal AF.^37^ In addition, inflammation has been proposed as a possible contributor to the development of thrombosis despite adequate oral anticoagulation.^38^ Inflammatory markers such as IL (interleukin)-6 and CRP (C-reactive protein) have been shown to be upregulated in blood samples taken during episodes of AF,^39^ and there is some evidence to suggest that the levels of cytokines such as TNF-α (tumor necrosis factor-α) vary according to AF subtype.^40^

Although the pattern of AF may be independently associated with recurrent ischemic stroke, patients with persistent AF also tend to have a higher burden of comorbidities, including previous stroke, hypertension, and heart failure. Indeed, a multicenter cohort study found that although patients with paroxysmal AF had a lower rate of early ischemic recurrence than patients with persistent AF, this was no longer the case after adjustment for risk factors for early ischemic recurrence.^18^

Our findings did not align with previous evidence suggesting that AF diagnosed before stroke onset is associated with a higher risk of stroke recurrence.^7,8^ However, AF diagnosed after stroke is increasingly discovered after prolonged cardiac monitoring, and this may be detected years after the index stroke if an implantable loop recorder is used.^41^ Within OPTIMAS, we could only enroll patients within the first few days from the index stroke and required 12 lead ECG evidence of AF if not already documented in the participant’s past medical history. Although 12-lead ECG-detected AF represents a new poststroke diagnosis, some studies have excluded these patients from the category of AF diagnosed after stroke because they probably have a higher burden AF with higher cardioembolic risk.^41^ It is therefore plausible that AF detected in the acute phase after an ischemic stroke (such as in OPTIMAS) conveys a higher cardioembolic risk than the cases of AF diagnosed after stroke included in previous literature, potentially contributing to the difference found in our study.

This subgroup analysis of the OPTIMAS trial has several strengths. We included a large sample size recruited across multiple sites, including a substantial proportion with moderate-to-severe stroke (42.5%), increasing its generalizability. However, the average age in OPTIMAS was 78 (SD, 9.9), and it may be that in younger cohorts with a lower burden of medical comorbidities, there is not the same observed difference in outcomes between AF subtypes. Our composite primary outcome—which assesses recurrent stroke, ICH, and systemic embolism—was independently adjudicated to provide a comprehensive measure of treatment efficacy and safety. The size of the trial allowed us to adjust for numerous potential confounding factors, including coexisting vascular risk factors, which could influence prognosis for the outcomes of interest.

We acknowledge some limitations. The subgroup analysis of AF subtype and our prognostic analyses were not prespecified in the main trial statistical analysis plan, and these analyses are exploratory. Nonetheless, our results provide clinicians with reassurance the early DOAC initiation is safe regardless of AF time of diagnosis and AF subtype in most cases. The categorization of AF subtype was adjudicated locally by research practitioners according to ECG or medical history, so some participants might have been misclassified. However, all practitioners were trained, and we do not expect any systematic bias in classification that could have affected our results. Although we collected a binary variable assessing the timing of AF diagnosis, data on the exact duration or burden of AF were not collected.^42^ Furthermore, AF is a dynamic entity with possible progression over time from paroxysmal to permanent subtypes.^43^ We did not collect data on whether those with paroxysmal AF transitioned into a permanent rhythm during the OPTIMAS trial. These could be important factors in determining future stroke risk and warrant specific studies aimed at addressing this question.

Although there were differences in some of the baseline characteristics between groups, with those in persistent AF being older with a higher prevalence of hypertension, higher baseline modified Rankin Scale score, and higher NIHSS score (Table S2), we mitigated the risk of confounding by adjusting for the presence of confounders. We were able to adjust for a large number of known risk factors for ischemic stroke in the context of AF. Nevertheless, it is possible that residual confounding from unmeasured factors, such as the severity of comorbidities like hypertension and diabetes, could have influenced our observations. Furthermore, we did not collect data on socioeconomic factors, which have been associated with differences in stroke outcomes.^44^ Another limitation of our study is the inclusion of relatively few participants of Black, Asian, and other non-White ethnicities, partly because of the characteristics of an older UK population with AF. This is relevant as there are recognized ethnic differences between Asians and Europeans for ischemic stroke and ICH.^45^ Finally, AF can be further classified into a permanent rhythm if previous attempts to restore sinus rhythm have been unsuccessful and no further attempts at restoration of sinus rhythm are planned.^20^ We did not include separate categories for persistent and permanent AF rhythms, which could have affected the findings.

## Conclusions

We found no evidence for heterogeneity in the effects of early DOAC treatment according to AF time of diagnosis or subtype within the OPTIMAS clinical trial, suggesting that DOAC treatment can be safely started early regardless of AF time of diagnosis or subtype, which is reassuring for clinicians. We found that persistent AF was independently associated with a higher risk of recurrent ischemic stroke compared with paroxysmal AF in adjusted analyses. Those with persistent AF might benefit from more intensive preventative treatment options, such as left atrial appendage occlusion, an approach which could be investigated with future randomized trials.

## Article Information

### Acknowledgments

The authors thank all the participants and their relatives or carers who generously gave their time to contribute to this research. The authors also thank their hospital doctors; their primary care practitioners; the trial steering committee; the independent data monitoring committee; and the independent external event adjudication committee. The authors also thank all of the OPTIMAS (Optimal Timing of Anticoagulation After Acute Ischemic Stroke) investigators.

### Disclosures

Dr Werring reports consulting fees from Novo Nordisk, National Institute for Health and Clinical Excellence, and Alnylam; payments or speaker honoraria from Novo Nordisk, Bayer, and AstraZeneca/Alexion; participation on a data safety monitoring board for the OXHARP trial (Oxford Haemodynamic Adaptation to Reduce Pulsatility), participation as Steering Committee Chair for the MACE-ICH (Mannitol for Cerebral Oedema After Intracerebral Haemorrhage) and PLINTH (Platform Study for Intracerebral Haemorrhage) trials; serving as President of the British and Irish Association of Stroke Physicians; and holding a National Institute for Health and Care Research Senior Investigator Award. Dr Norrving reports payments for work in a data safety monitoring board in the HOVID trial (Neuroprotective and Cardioprotective Effects of Palm Vitamin E Tocotrienols) and fees from Simbec Orion. Dr James reports travel or speaker honoraria from Daiichi Sankyo, Portola, and Boehringer Ingelheim. Dr Cohen reports speaker honoraria from Technoclone (paid to University College London Hospitals Charity) and GlaxoSmithKline; consulting fees from Union Chimique Belge Biopharma (paid to University College London Hospitals Charity); and advisory board fees from Roche and Argenx. Dr Lip reports being a consultant and speaker for Bristol-Myers Squibb/Pfizer, Boehringer Ingelheim, Daiichi-Sankyo, and Anthos (no fees received personally), and is a National Institute for Health and Care Research Senior Investigator. Dr Hunter reports grants from the National Institute for Health and Care Research for the Dementia Policy Research Unit—Queen Mary and the Research Support Service, and being co-chair of the European Union Transforming Health and Care Systems funding board in 2023. Dr Ford reports receiving consulting fees from AstraZeneca for a project on management of stroke due to intracerebral hemorrhage (payment to his employer) and Bayer (for lecture on models of the National Health Service industry working) and is Chief Executive of Health Innovation (Oxford and Thames Valley), which has multiple joint working agreements and medical education grants with industry partners that have contracts with Oxford University Hospitals NHS (National Health Service) Trust. Dr Freemantle reports consulting fees received from ALK (Allergologisk Laboratorium København), Sanofi Aventis, Gedeon Richter, Abbot, Galderma, AstraZeneca, Ipsen, Vertex, Thea, Novo Nordisk, Aimmune, and Gilead. The other authors report no conflicts.

### Supplemental Material

Tables S1–S3

CONSORT Checklist

## APPENDIX

B. Jelley (PI), T. Hughes, M. Evans, D.G. Esteban, L. Knibbs, L. Broad, R. Price, L.H. Griebel, S. Hewson, K. Thavanesan (PI), L. Mallon, A. Smith, M. White, L. Zhang (PI), B. Clarke, Y. Abousleiman, L. Binnie, C.H. Sim, M. Castanheira, P. Ferdinand (PI), R. Varquez, I. Ponce, S. Saxena, E. O’Brien (PI), J.D. Reyes, J. Mitchell-Douglas, J. Francis, S. Banerjee (PI), V. Dave, S. Mashate, T. Patel, L. Sekaran (PI), W. Murad, A. Asaipillai, S. Sakthivel, T.L. Margaret, J. Angus, L. Reid, C. Fornolles, S. Sundayi, L. Poolon, F. Justin, S. Hunte, M. Bhandari (PI), S. Sundayi, J. Kho, V. Cvoro (PI), R. Parakramawansha, M. Couser, H. Hughes, A. Naqvi (PI), K. Harkness, E. Richards, J. Howe, C. Kamara, J. Gardner, H. Bains (PI), R. Teal, J. Joseph, J. Benjamin, S. Al-Hussayni (PI), G. Thomas, F. Robinson, L. Dixon, M. Krishnan (PI), P. Slade, T. Anjum, S. Storton, K. Adie (PI), K. Northcott, K. Morgan, E. Williams, H. Chandrashekar (PI), H. Maguire, C. Gabriel, D. Maren, H. David, S. Clarke, K. Nagaratnam (PI), V. Nelatur, N. Mannava, L. Blasco, J. Devine (PI), R. Bathula, P. Gopi, N. Mehta, S. Sreedevi Raj, J. Teo (PI), L. Sztriha, Y. Mah, S. Ankolekar, B. Sari, M. Tibajai, A. Morgan, M. Recaman, S. Bayhonan, C. Belo, M. James (PI), S. Finch, S. Keenan, A. Bowring, A. Shetty (PI), S. Chan, L. Gray, T. Harrison (PI), O. Spooner, E. Kinsella-Perks, E. Erumere, B. Sanders, D. Sims (PI), M. Willmot, E. Littleton, E. Spruce, L. Moody, C. Sheriden, S. Luxmore-Brown, A. Neal, S. Beddows, M.A. Tuna (PI), A. Misra, R. Penn, S. Mariampillai, I. Anwar (PI), A. Annamalai, S. Whitehouse, L. Shepherd, E. Siddle, K. Chatterjee (PI), S. Leason, A. Davies, R. Marigold (PI), S. Frank, A. Baird, T. Hannam-Penfold, L. Inacio, S. Smith, D. Eveson (PI), K. Musarrat, S. Khan, T. Harris, M.R. Chowdhury (PI), S. Alam, E. Jamieson, E. Anyankpele, F. Al Shalchi, V. Rivers, S. Bell, R. Francis, D. Beeby, J. Finch, M.J. Macleod (PI), G. Guzman-Gutierrez, K. Carter, J. Irvine, L. Gbadamoshi (PI), T. Costa, S. Heirons, H. Stoney, L. Shaw, J. Choulerton, D Catibog, N Sattar (PI), M Myint, A Smith, K Serac, H Emsley (PI), C Anazodo, S Sultan, B Gregary, A Brown, A Mahmood (PI), N. Chattha, W. Old, C. Pegg, M. Davey, M. Page, B. Sandhu, E. Phiri, K. Rashed (PI), E. Wilson, E. Hindley, S. Board, S. Antony, A. Tanate, M. Davis (PI), A. Dixit, V. Slater, M. Fawcett, T. England (PI), J. Scott, J. Beavan, A. Hedstrom, D. Karunatilake (PI), K. Gillmain, N. Singh, T. Hallows, M. Barber (PI), L. Yates, C. Micallef, D. Esson, Wai Meng Yu (PI), B. Jaa Ming New, A. Matos, C. Burt, L. Cabrelli, G. Wilkie, M. Meegada (PI), R. Kirthivasan, C. Fox, V. Mead, A. Lyle, R. Saksena (PI), A. Bakshi, A. O’Kelly, J. Rehan (PI), O. Ebueka, M. Cooper, I. Wynter, S. Smith, S. Kumar (PI), L. O’Brien, Cerrys Parker, Emma Parker, N. Khan (PI), C. Patterson, S. Maguire, O. Quinn, R. Bellfield, Y. Behnam (PI), J. Costa, C. Padilla-Harris, L. Moram, S.A. Raza (PI), H. Tench, T. Sims, H. McGuinness, R. Loosley, R. Wolf-Roberts, S. Buddha (PI), I. Salt, K. Lewis, S. Mavinamne (PI), C. Ditchfield, S. Dealing, A. Shah (PI), G. Crossingham, M. Mwadeyi, A. Kenton (PI), F. Omoregie, D. Hargroves (PI), S. Abubakar, A. Warwick, G. Hector, S. Maguire (PI), Hassan, E. Veraque, M. Farman, L. Makawa, A. Byrne (PI), J. Kirkham, G. Blayney, J. Selwyn, P. Kakar (PI), M. Al Khaddour, R. Dhami, E. Baker, B. Esisi (PI), E. Clarkson, D. Fellowes, J. Kresmir (PI), P. Guyler, D. Ngo, I. Wijenayake, S. Tysoe, J. Galliford, P. Harman, M. Garside (PI), M. Badanahatti, A. Smith, V. Riddell, G. Gramizadeh (PI), D. Dutta, M. Bajoriene, H. Erdogan, D. Ward, F. Doubal (PI), N. Samarasekera, S. Risbridger, A. MacRaild, A. Azim (PI), L. Wood, R. Tempest, R. Shekhar (PI), U. Rai, T. Fuller, A. Joshy, E. Nadar, M. Kini (PI), S. Ahmad, M. Robinson, L. King, V. Srinivasan (PI), M. Karwacka-Cichomska, V. Moore, K. Smith, B. Kariyadil, K. Kong (PI), K. Jergovic, K. Hubbard, S. Arif, M. Hasan (PI), N. Temple, D. Arcoria, Z. Horne, T. Soe (PI), H. Wyllie, C. Hacon, H. Sutherland, B. Menezes (PI), V. Johnson, N. Smyth (PI), Z. Mehdi, E. Tone, A. Bradley, E. Levell, A Ekkert (PI), S. Mazzucco, L. McCafferty, L. Vonoven, S. Dewan, P. Sridhar (PI), J. Thomas, S. Coetzee, B. Icke, J. Williams, N. Saravanan (PI), P. Bradley, R.M. Gibson, J. Antony, I. Ashraf (PI), J. Mabutti, C. Kamundi, P. Patiola, N. Oakley, H. Proeschel (PI), D. Keely, W. Longley, A. Cave, C. Ambrico, T. Black (PI), E. Porretta, A. Anthony, S. Ragab (PI), J. Dube, S. Kausar (PI), A. Gujjar, D.M. Abdullah, D. Kaur, N. Gadapa (PI), S. Choudhary, N. Nisar, G. Fawehinmi, K. Dunne, S. King, A. Kishore (PI), S. Lee, T. Marsden, M. Slaughter, K. Cawley, J. Perez, P. Anderton (PI), S. Soussi, D. Walstow, R. Pugh, A. Manoj (PI), G. Fletcher, P. Lopez, M. McCormick (PI), M. Magee, G. Tallon, D. McFarland, D. Cosgrove, N. Shinh (PI), K. Metcalf, A. Kostyuk, S. McDonald, S. Sayers, W. Sayed (PI), S. Abraham, G. Szabo, G. Crosbie, J. McIlmoyle (PI), P. Fearon, K. Courtney, S. Tauro, A. Singh (PI), A. Nair, S. Duberley, S. Philip, C. Curley, W. Goddard, Luke Bridge (PI), P. Wilcoxson, P. Wanklyn, J. Owen, J. France (PI), B. Reed, A. Foulds, B. Richard (PI), L. Parfitt, B. Affley (PI), C. Russo, M. Dsouza, E. Cruddas, D. Hargroves (PI), J. Rand, S. Shekar (PI), Y. Bhat, G. Marshall, M. Nash, N. Ahmad (PI), B.O. Okoko, R. Evans, T. Taylor, J. Dawson (PI), E. Colquhoun, C. James (PI), C. Aguirre, C. MacPhee, J. Phipps, S. Ispoglou (PI), A. Hayes, R. Evans.

## Supplementary Material

## References

[1] Benjamin EJ Wolf PA D’Agostino RB Silbershatz H Kannel WB Levy D. Impact of atrial fibrillation on the risk of death: the Framingham Heart Study. Circulation. 1998;98:946–952. doi: 10.1161/01.cir.98.10.946 9737513 10.1161/01.cir.98.10.946

[2] Wolf PA Abbott RD Kannel WB. Atrial fibrillation as an independent risk factor for stroke: the Framingham Study. Stroke. 1991;22:983–988. doi: 10.1161/01.str.22.8.983 1866765 10.1161/01.str.22.8.983

[3] Hart RG Pearce LA Aguilar MI. Meta-analysis: antithrombotic therapy to prevent stroke in patients who have nonvalvular atrial fibrillation. Ann Intern Med. 2007;146:857–867. doi: 10.7326/0003-4819-146-12-200706190-00007 17577005 10.7326/0003-4819-146-12-200706190-00007

[4] Ruff CT Giugliano RP Braunwald E Hoffman EB Deenadayalu N Ezekowitz MD Camm AJ Weitz JI Lewis BS Parkhomenko A. Comparison of the efficacy and safety of new oral anticoagulants with warfarin in patients with atrial fibrillation: a meta-analysis of randomised trials. Lancet. 2014;383:955–962. doi: 10.1016/S0140-6736(13)62343-0 24315724 10.1016/S0140-6736(13)62343-0

[5] De With RR Erkuner O Rienstra M Nguyen BO Korver FWJ Linz D Cate TH Spronk H Kroon AA Maass AH. Temporal patterns and short-term progression of paroxysmal atrial fibrillation: data from RACE V. Europace. 2020;22:1162–1172. doi: 10.1093/europace/euaa123 32642768 10.1093/europace/euaa123 PMC7400474

[6] Packer DL Mark DB Robb RA Monahan KH Bahnson TD Poole JE Noseworthy PA Rosenberg YD Jeffries N Mitchell LB; CABANA Investigators. Effect of catheter ablation vs antiarrhythmic drug therapy on mortality, stroke, bleeding, and cardiac arrest among patients with atrial fibrillation: the CABANA randomized clinical trial. JAMA. 2019;321:1261–1274. doi: 10.1001/jama.2019.0693 30874766 10.1001/jama.2019.0693 PMC6450284

[7] Sposato LA Field TS Schnabel RB Wachter R Andrade JG Hill MD. Towards a new classification of atrial fibrillation detected after a stroke or a transient ischaemic attack. Lancet Neurol. 2024;23:110–122. doi: 10.1016/S1474-4422(23)00326-5 37839436 10.1016/S1474-4422(23)00326-5

[8] Fridman S Jimenez-Ruiz A Vargas-Gonzalez JC Sposato LA. Differences between atrial fibrillation detected before and after stroke and TIA: a systematic review and meta-analysis. Cerebrovasc Dis. 2022;51:152–157. doi: 10.1159/000520101 34844239 10.1159/000520101

[9] Al-Khatib SM Thomas L Wallentin L Lopes RD Gersh B Garcia D Ezekowitz J Alings M Yang H Alexander JH. Outcomes of apixaban vs. warfarin by type and duration of atrial fibrillation: results from the ARISTOTLE trial. Eur Heart J. 2013;34:2464–2471. doi: 10.1093/eurheartj/eht135 23594592 10.1093/eurheartj/eht135

[10] Bansilal S Bloomgarden Z Halperin JL Hellkamp AS Lokhnygina Y Patel MR Becker RC Breithardt G Hacke W Hankey GJ; ROCKET AF Steering Committee and Investigators. Efficacy and safety of rivaroxaban in patients with diabetes and nonvalvular atrial fibrillation: the Rivaroxaban Once-daily, Oral, Direct Factor Xa Inhibition Compared with Vitamin K Antagonism for Prevention of Stroke and Embolism Trial in Atrial Fibrillation (ROCKET AF Trial). Am Heart J. 2015;170:675.e8–682.e8. doi: 10.1016/j.ahj.2015.07.006 26386791 10.1016/j.ahj.2015.07.006

[11] Eisen A Giugliano RP Ruff CT Nordio F Gogia HS Awasty VR Henderson DA Mercuri MF Rutman H Antman EM. Edoxaban vs warfarin in patients with nonvalvular atrial fibrillation in the US Food and Drug Administration approval population: an analysis from the Effective Anticoagulation with Factor Xa Next Generation in Atrial Fibrillation-Thrombolysis in Myocardial Infarction 48 (ENGAGE AF-TIMI 48) trial. Am Heart J. 2016;172:144–151. doi: 10.1016/j.ahj.2015.11.004 26856226 10.1016/j.ahj.2015.11.004

[12] Banerjee A Taillandier S Olesen JB Lane DA Lallemand B Lip GY Fauchier L. Pattern of atrial fibrillation and risk of outcomes: the Loire Valley Atrial Fibrillation Project. Int J Cardiol. 2013;167:2682–2687. doi: 10.1016/j.ijcard.2012.06.118 22795403 10.1016/j.ijcard.2012.06.118

[13] Disertori M Franzosi MG Barlera S Cosmi F Quintarelli S Favero C Cappellini G Fabbri G Maggioni AP Staszewsky L; GISSI-AF investigators. Thromboembolic event rate in paroxysmal and persistent atrial fibrillation: data from the GISSI-AF trial. BMC Cardiovasc Disord. 2013;13:28. doi: 10.1186/1471-2261-13-28 23586654 10.1186/1471-2261-13-28 PMC3639147

[14] Inoue H Atarashi H Okumura K Yamashita T Kumagai N Origasa H. Thromboembolic events in paroxysmal vs. permanent non-valvular atrial fibrillation. Subanalysis of the J-RHYTHM registry. Circ J. 2014;78:2388–2393. doi: 10.1253/circj.cj-14-0507 25099606 10.1253/circj.cj-14-0507

[15] Nieuwlaat R Dinh T Olsson SB Camm AJ Capucci A Tieleman RG Lip GY Crijns HJ Euro Heart Survey I. Should we abandon the common practice of withholding oral anticoagulation in paroxysmal atrial fibrillation? Eur Heart J. 2008;29:915–922. doi: 10.1093/eurheartj/ehn101 18334476 10.1093/eurheartj/ehn101

[16] Hohnloser SH Pajitnev D Pogue J Healey JS Pfeffer MA Yusuf S Connolly SJ Investigators AW. Incidence of stroke in paroxysmal versus sustained atrial fibrillation in patients taking oral anticoagulation or combined antiplatelet therapy: an ACTIVE W Substudy. J Am Coll Cardiol. 2007;50:2156–2161. doi: 10.1016/j.jacc.2007.07.076 18036454 10.1016/j.jacc.2007.07.076

[17] Ntaios G Sagris D Buckley BJR Harrison SL Abdul-Rahim A Austin P Lip GYH. Risk of myocardial infarction and ischemic stroke in individuals with first-diagnosed paroxysmal vs. non-paroxysmal atrial fibrillation under anticoagulation. Europace. 2023;25:euad143. doi: 10.1093/europace/euad143 37285483 10.1093/europace/euad143 PMC10246817

[18] Paciaroni M Angelini F Agnelli G Tsivgoulis G Furie KL Tadi P Becattini C Falocci N Zedde M Abdul-Rahim AH. Early recurrence in paroxysmal versus sustained atrial fibrillation in patients with acute ischaemic stroke. Eur Stroke J. 2019;4:55–64. doi: 10.1177/2396987318785853 31165095 10.1177/2396987318785853 PMC6533867

[19] Tomasdottir M Friberg L Hijazi Z Lindback J Oldgren J. Risk of ischemic stroke and utility of CHA2 DS2 -VASc score in women and men with atrial fibrillation. Clin Cardiol. 2019;42:1003–1009. doi: 10.1002/clc.23257 31490011 10.1002/clc.23257 PMC6788468

[20] Van Gelder IC Rienstra M Bunting KV Casado-Arroyo R Caso V Crijns HJ De Potter TJ Dwight J Guasti L Hanke T; ESC Scientific Document Group. [2024 ESC Guidelines for the management of atrial fibrillation developed in collaboration with the European Association for Cardio-Thoracic Surgery (EACTS)]. G Ital Cardiol (Rome). 2025;26:e1–e104. doi: 10.1714/4419.44150 10.1714/4419.44150 39898766

[21] Werring DJ Dehbi HM Ahmed N Arram L Best JG Balogun M Bennett K Bordea E Caverly E Chau M; OPTIMAS investigators. Optimal timing of anticoagulation after acute ischaemic stroke with atrial fibrillation (OPTIMAS): a multicentre, blinded-endpoint, phase 4, randomised controlled trial. Lancet. 2024;405:1731–1741. doi: 10.1016/S0140-6736(24)02197-4 10.1016/S0140-6736(24)02197-4 39491870

[22] Best JG Arram L Ahmed N Balogun M Bennett K Bordea E Campos MG Caverly E Chau M Cohen H; OPTIMAS investigators. Optimal timing of anticoagulation after acute ischemic stroke with atrial fibrillation (OPTIMAS): protocol for a randomized controlled trial. Int J Stroke. 2022;17:583–589. doi: 10.1177/17474930211057722 35018878 10.1177/17474930211057722

[23] Berger C Fiorelli M Steiner T Schabitz WR Bozzao L Bluhmki E Hacke W von Kummer R. Hemorrhagic transformation of ischemic brain tissue: asymptomatic or symptomatic? Stroke. 2001;32:1330–1335. doi: 10.1161/01.str.32.6.1330 11387495 10.1161/01.str.32.6.1330

[24] Electronic Medicines Compendium. Eliquis 5 mg film-coated tablets—summary of product characteristics. Accessed November 6, 2024. https://www.medicines.org.uk/emc/product/2878/smpc

[25] Electronic Medicines Compendium. Xarelto 20mg film-coated tablets—summary of product characteristics. Accessed November 6, 2024. https://www.medicines.org.uk/emc/product/2793/smpc

[26] Electronic Medicines Compendium. Lixiana 60mg film-coated tablets—summary of product characteristics. Accessed November 6, 2024. https://www.medicines.org.uk/emc/product/6905/smpc

[27] Electronic Medicines Compendium. Pradaxa 150 mg hard capsules—summary of product characteristics. Accessed November 6, 2024. https://www.medicines.org.uk/emc/product/4703/smpc

[28] Ahmed N Dehbi HM Freemantle N Best J Nash PS Ruffle JK Doig D Werring DJ. Optimal Timing of Anticoagulation After Acute Ischaemic Stroke With Atrial Fibrillation (OPTIMAS): statistical analysis plan for a randomised controlled trial. Trials. 2025;26:58. doi: 10.1186/s13063-025-08761-6 39966982 10.1186/s13063-025-08761-6 PMC11837694

[29] Harrell FE Jr Lee KL Mark DB. Multivariable prognostic models: issues in developing models, evaluating assumptions and adequacy, and measuring and reducing errors. Stat Med. 1996;15:361–387. doi: 10.1002/(SICI)1097-0258(19960229)15:4<361::AID-SIM168>3.0.CO;2-4 8668867 10.1002/(SICI)1097-0258(19960229)15:4<361::AID-SIM168>3.0.CO;2-4

[30] Chen Y Zhao Y Dang G Ouyang F Chen X Zeng J. Stroke event rates and the optimal antithrombotic choice of patients with paroxysmal atrial fibrillation: a systematic review and meta-analysis of randomized controlled trials. Medicine (Baltim). 2015;94:e2364. doi: 10.1097/MD.0000000000002364 10.1097/MD.0000000000002364 PMC5291617 26717376

[31] Chiang CE Naditch-Brule L Murin J Goethals M Inoue H O’Neill J Silva-Cardoso J Zharinov O Gamra H Alam S. Distribution and risk profile of paroxysmal, persistent, and permanent atrial fibrillation in routine clinical practice: insight from the real-life global survey evaluating patients with atrial fibrillation international registry. Circ Arrhythm Electrophysiol. 2012;5:632–639. doi: 10.1161/CIRCEP.112.970749 22787011 10.1161/CIRCEP.112.970749

[32] Deguchi I Hayashi T Fukuoka T Kobayashi S Tanahashi N; Japan Standard Stroke Registry Study Group. Features of cardioembolic stroke with persistent and paroxysmal atrial fibrillation—a study with the Japan Stroke Registry. Eur J Neurol. 2015;22:1215–1219. doi: 10.1111/ene.12728 25962447 10.1111/ene.12728

[33] Chao TF Potpara TS Lip GYH. Atrial fibrillation: stroke prevention. Lancet Reg Health Eur. 2024;37:100797. doi: 10.1016/j.lanepe.2023.100797 38362551 10.1016/j.lanepe.2023.100797 PMC10867001

[34] Maarse M Seiffge DJ Werring DJ Boersma LVA Beneduce A Tondo C Gasperetti A Aarnink EW Fierro N Mazzone P; RAF, RAF-DOAC; CROMIS-2; SAMURAI, NOACISP; Erlangen Registry; Verona Registry; STR-OAC LAAO Group. Left atrial appendage occlusion vs standard of care after ischemic stroke despite anticoagulation. JAMA Neurol. 2024;81:1150–1158. doi: 10.1001/jamaneurol.2024.2882 39374446 10.1001/jamaneurol.2024.2882 PMC11420820

[35] Ding WY Gupta D Lip GYH. Atrial fibrillation and the prothrombotic state: revisiting Virchow’s triad in 2020. Heart. 2020;106:1463–1468. doi: 10.1136/heartjnl-2020-316977 32675218 10.1136/heartjnl-2020-316977

[36] Gupta DK Shah AM Giugliano RP Ruff CT Antman EM Grip LT Deenadayalu N Hoffman E Patel I Shi M; Effective Anticoagulation With Factor Xa Next Generation in AF-Thrombolysis in Myocardial Infarction 48 Echocardiographic Study Investigators. Left atrial structure and function in atrial fibrillation: ENGAGE AF-TIMI 48. Eur Heart J. 2014;35:1457–1465. doi: 10.1093/eurheartj/eht500 24302269 10.1093/eurheartj/eht500 PMC4048534

[37] Kamath S Chin BS Blann AD Lip GY. A study of platelet activation in paroxysmal, persistent and permanent atrial fibrillation. Blood Coagul Fibrinolysis. 2002;13:627–636. doi: 10.1097/00001721-200210000-00008 12439149 10.1097/00001721-200210000-00008

[38] Singleton MJ Yuan Y Dawood FZ Howard G Judd SE Zakai NA Howard VJ Herrington DM Soliman EZ Cushman M. Multiple blood biomarkers and stroke risk in atrial fibrillation: the REGARDS study. J Am Heart Assoc. 2021;10:e020157. doi: 10.1161/JAHA.120.020157 34325516 10.1161/JAHA.120.020157 PMC8475705

[39] Ihara K Sasano T. Role of inflammation in the pathogenesis of atrial fibrillation. Front Physiol. 2022;13:862164. doi: 10.3389/fphys.2022.862164 35492601 10.3389/fphys.2022.862164 PMC9047861

[40] Wu L Emmens RW van Wezenbeek J Stooker W Allaart CP Vonk ABA van Rossum AC Niessen HWM Krijnen PAJ. Atrial inflammation in different atrial fibrillation subtypes and its relation with clinical risk factors. Clin Res Cardiol. 2020;109:1271–1281. doi: 10.1007/s00392-020-01619-8 32072262 10.1007/s00392-020-01619-8 PMC7515944

[41] Sposato LA Lip GYH Haeusler KG. Atrial fibrillation first detected after stroke: is timing and detection intensity relevant for stroke risk? Eur Heart J. 2024;45:396–398. doi: 10.1093/eurheartj/ehad744 38014646 10.1093/eurheartj/ehad744

[42] Doehner W Boriani G Potpara T Blomstrom-Lundqvist C Passman R Sposato LA Dobrev D Freedman B Van Gelder IC Glotzer TV. Atrial fibrillation burden in clinical practice, research, and technology development: a clinical consensus statement of the European Society of Cardiology Council on Stroke and the European Heart Rhythm Association. Europace. 2025;27:euaf019. doi: 10.1093/europace/euaf019 40073206 10.1093/europace/euaf019 PMC11901050

[43] de Vos CB Pisters R Nieuwlaat R Prins MH Tieleman RG Coelen RJ van den Heijkant AC Allessie MA Crijns HJ. Progression from paroxysmal to persistent atrial fibrillation clinical correlates and prognosis. J Am Coll Cardiol. 2010;55:725–731. doi: 10.1016/j.jacc.2009.11.040 20170808 10.1016/j.jacc.2009.11.040

[44] Marshall IJ Wang Y Crichton S McKevitt C Rudd AG Wolfe CD. The effects of socioeconomic status on stroke risk and outcomes. Lancet Neurol. 2015;14:1206–1218. doi: 10.1016/S1474-4422(15)00200-8 26581971 10.1016/S1474-4422(15)00200-8

[45] Kang DS Yang PS Kim D Jang E Yu HT Kim TH Sung JH Pak HN Lee MH Lip GYH. Racial differences in bleeding risk: an ecological epidemiological study comparing Korea and United Kingdom Subjects. Thromb Haemost. 2024;124:842–851. doi: 10.1055/a-2269-1123 38359877 10.1055/a-2269-1123 PMC11349425

