# Supplementary material for: Early Versus Delayed Anticoagulation in Acute Ischemic Stroke According to Atrial Fibrillation Subtype and Time of Diagnosis: Subgroup Analysis of the OPTIMAS Randomized Controlled Trial
Source: Stroke. 2026 Apr 1;57(6):1513–25. doi: 10.1161/STROKEAHA.125.055037 (PMC13196862; doi:10.1161/STROKEAHA.125.055037)
Supplement: Supplementary file 1 [file str-57-1513-s001.pdf]

SUPPLEMENTAL MATERIAL

Table S1. Complete list of OPTIMAS trial sites and investigators, listed in descending order of the number of participants recruited (quantified in brackets) with Principal Investigators denoted by PI

**University Hospital of Wales**, **Cardiff** (175) (Dr B Jelley (PI), Dr T Hughes, M Evans, D G Esteban, L Knibbs, L Broad, R Price, L H Griebel, S Hewson)
**Royal Bournemouth Hospital**, **Bournemouth** (166) (Dr K Thavanesan (PI), L Mallon, A Smith, M White)
**St Georges Hospital**, **London** (158) (Dr L Zhang (PI), Dr B Clarke, Dr Y Abousleiman, L Binnie, C H Sim, M Castanheira)
**University College London Hospitals NHS Foundation Trust, London** (147) (Dr F Humphries (PI), S Obarey, S Feerick, Y C Lee, A Lewis, R Muhammad, N Francia, N Atang, A Banaras, M Marinescu)
**Royal Stoke University Hospital, Stoke** (131) (Dr P Ferdinand (PI), R Varquez, I Ponce, S Saxena)
**Addenbrooke's Hospital, Cambridge** (111), (Dr E O'Brien (PI), Dr J D Reyes, J Mitchell-Douglas, J Francis)
**Charing Cross Hospital, London** (93) (Dr S Banerjee (PI), V Dave, S Mashate, T Patel)
**Luton & Dunstable University Hospital, Luton** (88) (Dr L Sekaran (PI), Dr W Murad, Dr A Asaipillai, Dr S Sakthivel, T L Margaret, J Angus, L Reid, C Fornolles, S Sundayi, L Poolon, F Justin, S Hunte)
**Watford General Hospital, Watford** (84) (Dr M Bhandari (PI), S Sundayi, J Kho)
**Victoria Hospital, Fife** (79) (Dr V Cvoro (PI), Dr R Parakramawansha, M Couser, H Hughes)
**Royal Hallamshire Hospital, Sheffield** (75) (Dr A Naqvi (PI), Dr K Harkness, E Richards, J Howe, C Kamara, J Gardner)
**John Radcliffe Hospital, Oxford** (75) (Dr H Bains (PI), R Teal, J Joseph, J Benjamin)
**James Cook University Hospital, Middlesborough** (73) (Dr S Al-Hussayni (PI), Dr G Thomas, F Robinson, L Dixon)
**Morriston Hospital, Swansea** (71) (Dr M Krishnan (PI), Dr P Slade, Dr T Anjum, S Storton)
**Royal Cornwall Hospital, Truro** (68) (Dr K Adie (PI), K Northcott, K Morgan, E Williams)
**Leighton Hospital, Crewe** (67) (Dr H Chandrashekar (PI), H Maguire, C Gabriel, D Maren, H David, S Clarke)
**Royal Berkshire, Reading** (66) (Dr K Nagaratnam (PI), Dr V Nelatur, N Mannava, L Blasco)
**Northwick Park Hospital, Harrow** (62) (Dr J Devine (PI), Dr R Bathula, P Gopi, N Mehta S Sreedevi Raj)
**King's College Hospital NHS Foundation Trust, London** (60) (Dr J Teo (PI), Dr L Sztriha, Dr Y Mah, Dr S Ankolekar, B Sari, M tibajai, A Morgan, M Recaman, S Bayhonan, C Belo)
**Royal Devon & Exeter Hospital, Exeter** (60) (Prof M James (PI), S Finch, S Keenan, A Bowring)
**Nottingham University Hospitals NHS Trust, Nottingham** (57) (Dr A Shetty (PI), Dr S Chan, L Gray)

**Royal London Hospital, London** (53) (Dr T Harrison (PI), Dr O Spooner, E Kinsella-Perks, E Erumere, B Sanders)
**Queen Elizabeth University Hospital, Birmingham** (48) (Dr D Sims (PI), Dr M Willmot, Dr E Littleton, E Spruce, L Moody, C Sheriden, S Luxmore-Brown, A Neal, S Beddows)
**Wycombe Hospital, High Wycombe** (45) (Dr M A Tuna (PI), Dr A Misra, R Penn, S Mariampillai)
**North Tees Hospital, Stockton-on-Tees** (45) (Dr I Anwar (PI), Dr A Annamalai, Dr S Whitehouse, L Shepherd, E Siddle)
**Countess of Chester Hospital, Chester** (44) (Dr K Chatterjee (PI), S Leason, A Davies)
**Southampton General Hospital, Southampton** (42) (Dr R Marigold (PI), S Frank, A Baird, T Hannam-Penfold, L Inacio, S Smith)
**Leicester Royal Infirmary, Leicester** (41) (Dr D Eveson (PI), Dr K Musarrat, S Khan, T Harris)
**Ipswich Hospital, Ipswich** (41) (Dr M R Chowdhury (PI), Dr S Alam, Dr E Jamieson, Dr E Anyankpele, Dr F Al Shalchi, V Rivers, S Bell, R Francis, D Beeby, J Finch)
**Aberdeen Royal Infirmary, Aberdeen** (41) (Prof M J Macleod (PI), Dr G Guzman-Gutierrez, Dr K Carter, J Irvine)
**Royal United Hospital, Bath** (40) (Dr L Gbadamoshi (PI), T Costa, S Heirons, H Stoney, L Shaw, J Choulerton, D Catibog)
**Sunderland Royal Hospital, Sunderland** (39) (Dr N Sattar (PI), Dr M Myint, A Smith, K Serac)
**Royal Preston Hospital, Preston** (38) (Dr H Emsley (PI), Dr C Anazodo, Dr S Sultan, B Gregary, A Brown)
**Maidstone Hospital, Maidstone** (38) (Dr A Mahmood (PI), Dr N Chattha, Dr W Old, C Pegg, M Davey, M Page, B Sandhu, E Phiri)
**Yeovil District Hospital, Yeovil** (37) (Dr K Rashed (PI), Dr E Wilson, Dr E Hindley, S Board, S Antony, A Tanate)
**Royal Victoria Infirmary, Newcastle** (37) (Dr M Davis (PI), Dr A Dixit, V Slater, M Fawcett)
**Royal Derby Hospital, Derby** (36) (Dr T England (PI), Dr J Scott, Dr J Beavan, A Hedstrom)
**Musgrove Park Hospital, Taunton** (36) (Dr D Karunatilake (PI), K Gillmain, N Singh, T Hallows)
**University Hospital Monklands, Airdrie** (36) (Dr M Barber (PI), Dr L Yates, Dr C Micallef, D Esson)
**Ninewells Hospital and Medical School, Dundee** (34) (Dr Wai Meng Yu (PI), Dr B Jaa Ming New, Dr A Matos, C Burt, L Cabrelli, G Wilkie)
**Broomfield Hospital, Chelmsford** (33) (Dr M Meegada (PI), Dr R Kirthivasan, C Fox, V Mead, A Lyle)
**Colchester General Hospital, Colchester** (32) (Dr R Saksena (PI), A Bakshi, A O'Kelly)
**Kings Mill Hospital, Sutton-in-Ashfield** (31) (Dr J Rehan (PI), Dr O Ebueka, Dr M Cooper, I Wynter, S Smith)
**Prince Philip Hospital, Llanelli** (31) (Dr S Kumar (PI), L O'Brien, Cerrys Parker, Emma Parker)
**Bradford Royal Infirmary, Bradford** (31) (Dr N Khan (PI), Dr C Patterson, Dr S Maguire, O Quinn, R Bellfield)

**Milton Keynes University Hospital, Milton Keynes** (29) (Dr Y Behnam (PI), Dr J Costa, C Padilla-Harris, L Moram)
**Bronglais General Hospital, Aberystwyth** (29) (Dr S A Raza (PI), H Tench, T Sims, H McGuinness, R Loosley, R Wolf-Roberts)
**Southmead Hospital, Bristol** (29) (Dr S Buddha (PI), Dr I Salt, K Lewis)
**Whiston Hospital, Prescot** (28) (Dr S Mavinamne (PI), C Ditchfield, S Dealing)
**Derriford Hospital, Plymouth** (27) (Dr A Shah (PI), Dr G Crossingham, M Mwadeyi)
**University Hospital of Coventry, Coventry** (27) (Dr A Kenton (PI), F Omoregie)
**Kent and Canterbury Hospital, Canterbury** (27) (Dr D Hargroves (PI), Dr S Abubakar, A Warwick, G Hector)
**Leeds General Infirmary, Leeds** (26) (Dr S Maguire (PI), Dr Hassan, E Veraque, M Farman, L Makawa)
**Forth Valley Royal Hospital, Larbert** (26) (Dr A Byrne (PI), Dr J Kirkham, Dr G Blayney, Prof J Selwyn)
**Epsom General Hospital, Epsom** (26) (Dr P Kakar (PI), Dr M Al Khaddour, R Dhami, E Baker)
**Hull Royal Infirmary, Hull** (25) (Dr B Esisi (PI), E Clarkson, D Fellowes)
**Southend Hospital, Southend** (24) (Dr J Kresmir (PI), Dr P Guyler, Dr D Ngo, Dr I Wijenayake, S Tysoe, J Galliford, P Harman)
**Northumbria (Hexham, North Tyneside, Wansbeck), Cramlington** (24) (Dr M Garside (PI), Dr M Badanahatti, A Smith, V Riddell)
**Gloucestershire Royal Hospital, Gloucester** (24) (Dr G Gramizadeh (PI), Dr D Dutta, Dr M Bajoriene, Dr H Erdogan, D Ward)
**Royal Infirmary of Edinburgh, Edinburgh** (20) (Dr F Doubal (PI), Dr N Samarasekera, S Risbridger, A MacRaild)
**West Suffolk Hospital, Bury-St-Edmunds** (19) (Dr A Azim (PI), L Wood, R Tempest)
**Queen Elizabeth Hospital, King's Lynn (19)** (Dr R Shekhar (PI), Dr U Rai, T Fuller, A Joshy, E Nadar)
**Calderdale and Huddersfield NHS Foundation Trust, Halifax** (19) (Dr M Kini (PI), Dr S Ahmad, M Robinson, L King)
**Northampton General Hospital, Northampton** (19) (Dr V Srinivasan (PI), Dr M Karwacka-Cichomska, V Moore, K Smith, B Kariyadil)
**Lincoln County Hospital, Lincoln** (17) (Dr K Kong (PI), Dr K Jergovic, K Hubbard, S Arif)
**Peterborough City Hospital, Peterborough** (17) (Dr M Hasan (PI), N Temple, D Arcoria, Z Horne)
**James Paget University Hospital, Great Yarmouth** (16) (Dr T Soe (PI), Dr H Wyllie, C Hacon, H Sutherland)
**Arrowe Park Hospital, Birkenhead** (15) (Dr B Menezes (PI), V Johnson)
**Royal Hampshire County Hospital, Winchester** (14) (Dr N Smyth (PI), Dr Z Mehdi, Dr E Tone, A Bradley, E Levell)
**Great Western Hospital, Swindon** (14) (Dr A Ekkert (PI), Dr S Mazzucco, L McCafferty. L Vonoven, S Dewan)
**Glangwili General Hospital, Carmarthen** (13) (Dr P Sridhar (PI), J Thomas, S Coetzee, B Icke, J Williams)

**Fairfield General Hospital, Bury** (13) (Dr N Saravanan (PI), P Bradley, R M Gibson, J Antony)
**Darent Valley Hospital, Dartford** (13) (Dr I Ashraf (PI), J Mabutti, C Kamundi, P Patiola, N Oakley)
**Dorset County Hospital, Dorchester** (12) (Dr H Proeschel (PI), Dr D Keely, W Longley, A Cave, C Ambrico)
**Salisbury District Hospital, Salisbury** (11) (Dr T Black (PI), Dr E Porretta, A Anthony)
**Poole Hospital, Poole** (11) (Dr S Ragab (PI), J Dube)
**Russell's Hall Hospital, Dudley** (11) (Dr S Kausar (PI), Dr A Gujjar, D M Abdullah, D Kaur)
**Queen's Hospital, Romford** (10) (Dr N Gadapa (PI), Dr S Choudhary, Dr N Nisar, G Fawehinmi, K Dunne, S King)
**Salford Royal Hospital, Salford** (10) (Dr A Kishore (PI), S Lee, T Marsden, M Slaughter, K Cawley, J Perez)
**Doncaster Royal Infirmary, Doncaster** (10) (Dr P Anderton (PI), Dr S Soussi, D Walstow, R Pugh)
**Royal Liverpool Hospital, Liverpool** (9) (Dr A Manoj (PI), G Fletcher, P Lopez)
**Craigavon Area Hospital, Portadown** (9) (Dr M McCormick (PI), Dr M Magee, Dr G Tallon, D McFarland, D Cosgrove)
**Norfolk and Norwich University Hospital, Norwich** (9) (Dr N Shinh (PI), Dr K Metcalf, Dr A Kostyuk, S McDonald, S Sayers)
**Wrexham Maelor Hospital, Wrexham** (8) (Dr W Sayed (PI), Dr S Abraham, G Szabo, G Crosbie)
**Royal Victoria Hospital, Belfast** (6) (Dr J McIlmoyle (PI), Dr P Fearon, K Courtney, S Tauro)
**Royal Blackburn Hospital, Blackburn** (6) (Dr A Singh (PI), Dr A Nair, S Duberley, S Philip, C Curley, W Goddard)
**York General Hospital, York** (5) (Dr Luke Bridge (PI), Dr P Wilcoxson, Dr P Wanklyn, J Owen)
**Torbay Hospital, Torquay** (5) (Dr J France (PI), B Reed, A Foulds)
**Nevill Hall Hospital, Abergavenny** (5) (Dr B Richard (PI), L Parfitt)
**St. Peter's Hospital, Chertsey** (4) (Dr B Affley (PI), Dr C Russo, M Dsouza, E Cruddas)
**William Harvey Hospital, Ashford** (3) (Dr D Hargroves (PI), J Rand)
**Royal Gwent Hospital - The Grange, Newport** (3) (Dr S Shekar (PI), Dr Y Bhat, G Marshall, M Nash)
**New Cross Hospital, Wolverhampton** (3) (Dr N Ahmad (PI), B O Okoko, R Evans, T Taylor)
**Queen Elizabeth University Hospital, Glasgow** (2) (Dr J Dawson (PI), E Colquhoun)
**Withybush General Hospital, Haverford West** (1) (Dr C James (PI), Dr C Aguirre, C MacPhee, J Phipps)
**Sandwell General Hospital, West Bromwich** (1) (Dr S Ispoglou (PI), A Hayes, R Evans)

Abbreviations: PI = Principle investigator, NHS = National Health Service

Table S2. Baseline characteristics according to AF subtype

Atrial Fibrillation Subtype	Paroxysmal (n=966)	Persistent (n=2653)	All participants (n=3619)	p-value
Age; years, mean (SD)	76.4 (10.5)	78.5 (9.6)	78.0 (9.9)	<0.001
Female sex	455 (47.1%)	1185 (44.7%)	1640 (45.3%)	0.193
Ethnicity				0.198
White	914 (94.6%)	2477 (93.4%)	3391 (93.7%)	
Black British; African or Caribbean	9 (0.9%)	49 (1.8%)	58 (1.6%)	
South Asian	19 (2.0%)	41 (1.5%)	60 (1.7%)	
East Asian or Southeast Asian	8 (0.8%)	32 (1.2%)	40 (1.1%)	
Mixed ethnicity; other; not disclosed or missing	16 (1.7%)	54 (2.0%)	70 (1.9%)	
Duration of AF				<0.001
Newly diagnosed	539 (55.8%)	1242 (46.8%)	1781 (49.2%)	
Known prior to stroke	427 (44.2%)	1411 (53.2%)	1838 (50.8%)	
Hypertension	616 (63.8%)	1818 (68.5%)	2434 (67.3%)	0.014
Diabetes	183 (18.9%)	585 (22.1%)	768 (21.2%)	0.07
Hypercholesterolemia	327 (33.9%)	861 (32.5%)	1188 (32.8%)	0.330
Chronic kidney disease	136 (14.1%)	407 (15.3%)	543 (15.0%)	0.348
Myocardial infarction	89 (9.2%)	247 (9.3%)	336 (9.3%)	0.372
History of angina	85 (8.8%)	177 (6.7%)	262 (7.2%)	0.025
Coronary revascularisation	70 (7.2%)	159 (6.0%)	229 (6.3%)	0.091
Congestive heart failure	85 (8.8%)	298 (11.2%)	383 (10.6%)	0.068
Peripheral arterial disease	23 (2.4%)	55 (2.1%)	78 (2.2%)	0.807
Previous ischemic stroke	140 (14.5%)	397 (15.0%)	537 (14.8%)	0.724
Previous other intracranial bleeding	19 (2.0%)	44 (1.7%)	63 (1.7%)	0.530
Known cognitive impairment	57 (5.9%)	191 (7.2%)	248 (6.9%)	0.092
Current smoker	81 (8.4%)	192 (7.2%)	273 (7.5%)	0.347
Previous smoker	256 (30.0%)	762 (32.5%)	1018 (31.8%)	0.078
Alcohol intake >14 units per week	95 (9.8%)	306 (11.5%)	401 (11.1%)	0.095
Previous anticoagulation	258 (26.7%)	1022 (38.5%)	1280 (35.4%)	<0.001
Anticoagulant drug type				0.051
Vitamin K antagonist	15 (1.6%)	99 (2.7%)	114 (3.1%)	
Direct oral anticoagulant	243 (25.2%)	923 (25.5%)	1166 (32.2%)	
Previous antiplatelet use	131 (13.6%)	276 (10.4%)	407 (11.2%)	0.008
IV Thrombolysis	246 (25.5%)	551 (20.8%)	797 (22.0%)	0.003
Endovascular treatment	70 (7.2%)	196 (7.4%)	266 (7.4%)	0.885
NIHSS on admission, median (IQR)	5.0 (3.0 to 10.0)	6.0 (3.0 to 11.0)	5.0 (3.0 to 10.0)	0.001
NIHSS at randomisation				<0.001
0-4	624 (64.6%)	1466 (55.3%)	2090 (57.8%)	
5-10	238 (24.6%)	767 (28.9%)	1005 (27.8%)	

11-15	55 (5.7%)	224 (8.4%)	279 (7.7%)	
16-21	39 (4.0%)	141 (5.3%)	180 (5.0%)	
>21	10 (1.0%)	55 (2.1%)	65 (1.8%)	
NIHSS at randomisation, median (IQR)	3.0 (1.0 to 6.0)	4.0 (2.0 to 8.0)	4.0 (2.0 to 7.0)	<0.001
Systolic BP mmHg, mean (SD)	134.1 (19.5)	134.5 (19.1)	134.4 (19.2)	0.640
Diastolic BP mmHg, mean (SD)	75.4 (12.4)	76.9 (13.0)	76.5 (12.8)	0.002
Pre-Stroke mRS,				0.002
No symptoms	544 (56.4%)	1291 (48.7%)	1835 (50.8%)	
No significant disability	191 (19.8%)	560 (21.1%)	751 (20.8%)	
Slight disability	98 (10.2%)	335 (12.6%)	433 (12.0%)	
Moderate disability	90 (9.3%)	326 (12.3%)	416 (11.5%)	
Moderately severe disability	37 (3.8%)	124 (4.7%)	161 (4.5%)	
Severe disability	5 (0.5%)	14 (0.5%)	19 (0.5%)	
Pre-Stroke mRS, median (IQR)	0 (0-1)	1 (0 -2)	0 (0-2)	0.002

Abbreviations: SD = standard deviation, AF = atrial fibrillation, IV = intravenous, NIHSS = NIH stroke scale, IQR = interquartile range, BP = blood pressure, mmHg = millimetres of mercury, mRS = modified Rankin score

Table S3. Univariable and multivariable mixed effects logistic regression models showing predictors of the composite primary outcome according to AF time of diagnosis

	Univariable		Multivariable model 1		Multivariable model 2	
**Predictor**	**Odds ratio (95% CI)**	**p-value**	**Odds ratio (95% CI)**	**p-value**	**Odds ratio (95% CI)**	**p-value**
Timing of AF diagnosis		0.098		0.877		0.600
After stroke	Ref.		Ref.		Ref.	
Before stroke	1.31 (0.94 – 1.99)		1.03 (0.68 – 1.58)		1.11 (0.75 – 1.65)	
Age	1.02 (1.00 – 1.04)	0.042	1.02 (1.00 – 1.04)	0.109	1.01 (0.99 – 1.04)	0.183
Female sex	1.27 (0.88 – 1.83)	0.207	1.33 (0.88 – 2.00)	0.171	1.19 (0.81 – 1.74)	0.377
Early DOAC initiation	0.99 (0.69 – 1.43)	0.962	1.07 (0.72 – 1.58)	0.748	0.99 (0.68 – 1.44)	0.965
Hypertension	1.13 (0.75 – 1.69)	0.557	0.89 (0.58 – 1.38)	0.604		
Diabetes	1.46 (0.96 – 2.20)	0.074	1.22 (0.76 – 1.95)	0.410		
Hypercholesterolemia	1.06 (0.71 – 1.57)	0.779	0.92 (0.59 – 1.43)	0.703		
Ischaemic heart disease	1.14 (0.70 – 1.85)	0.603	0.90 (0.51 – 1.58)	0.706		
Congestive heart failure	1.55 (0.92 – 2.60)	0.097	1.11 (0.61 – 2.04)	0.729		
Previous stroke	1.70 (1.09 – 2.65)	0.018	1.36 (0.81 – 2.27)	0.247	1.52 (0.96 – 2.4)	0.074
Current smoker	1.02 (0.51 – 2.04)	0.966	1.27 (0.62 – 2.63)	0.516		
IV Thrombolysis	0.47 (0.26 – 0 .82)	0.008	0.53 (0.29 – 0.98)	0.044	0.52 (0.29 – 0.93)	0.028
Stroke severity (NIHSS) at randomisation		0.596		0.826		0.689
0 – 4	Ref.		Ref.		Ref.	
5 – 10	1.33 (0.88 – 2.00)		1.16 (0.74 – 1.83)		1.26 (0.83 – 1.90)	
11 – 15	1.21 (0.61 – 2.39)		1.25 (0.62 – 2.53)		1.12 (0.56 – 2.23)	
16 – 21	0.75 (0.27 – 2.09)		0.77 (0.27 – 2.19)		0.68 (0.24 – 1.91)	
>21	1.52 (0.46 – 5.05)		1.59 (0.46 – 5.45)		1.43 (0.43 – 4.78)	

Abbreviations: AF = atrial fibrillation, IV = intravenous, NIHSS = NIH stroke scale

Section/topic	No	CONSORT 2025 checklist item description	Reported on page no.
**Title and abstract**			
Title and structured abstract	1a	Identification as a randomised trial	1
	1b	Structured summary of the trial design, methods, results, and conclusions	3-4
**Open science**			
Trial registration	2	Name of trial registry, identifying number (with URL) and date of registration	3
Protocol and statistical analysis plan	3	Where the trial protocol and statistical analysis plan can be accessed	8, 11
Data sharing	4	Where and how the individual de-identified participant data (including data dictionary), statistical code and any other materials can be accessed	8
Funding and conflicts of interest	5a	Sources of funding and other support (eg, supply of drugs), and role of funders in the design, conduct, analysis and reporting of the trial	25
	5b	Financial and other conflicts of interest of the manuscript authors	25-26
**Introduction**			
Background and rationale	6	Scientific background and rationale	6-7
Objectives	7	Specific objectives related to benefits and harms	7-8
**Methods**			
Patient and public involvement	8	Details of patient or public involvement in the design, conduct and reporting of the trial	NA
Trial design	9	Description of trial design including type of trial (eg, parallel group, crossover), allocation ratio, and framework (eg, superiority, equivalence, non-inferiority, exploratory)	8
Changes to trial protocol	10	Important changes to the trial after it commenced including any outcomes or analyses that were not prespecified, with reason	NA
Trial setting	11	Settings (eg, community, hospital) and locations (eg, countries, sites) where the trial was conducted	8
Eligibility criteria	12a	Eligibility criteria for participants	8-9
	12b	If applicable, eligibility criteria for sites and for individuals delivering the interventions (eg, surgeons, physiotherapists)	NA
Intervention and comparator	13	Intervention and comparator with sufficient details to allow replication. If relevant, where additional materials describing the intervention and comparator (eg, intervention manual) can be accessed	8-10
Outcomes	14	Prespecified primary and secondary outcomes, including the specific measurement variable (eg, systolic blood pressure), analysis metric (eg, change from baseline, final value, time to event), method of aggregation (eg, median, proportion), and time point for each outcome	10-11
Harms	15	How harms were defined and assessed (eg, systematically, non-systematically)	10

Sample size	16a	How sample size was determined, including all assumptions supporting the sample size calculation	Detailed in main trial report
	16b	Explanation of any interim analyses and stopping guidelines	Detailed in main trial report
Randomisation:			9
Sequence generation	17a	Who generated the random allocation sequence and the method used	
	17b	Type of randomisation and details of any restriction (eg, stratification, blocking and block size)	10
			**Reported on page no.**
Allocation concealment mechanism	18	Mechanism used to implement the random allocation sequence (eg, central computer/telephone; sequentially numbered, opaque, sealed containers), describing any steps to conceal the sequence until interventions were assigned	10
Implementation	19	Whether the personnel who enrolled and those who assigned participants to the interventions had access to the random allocation sequence	10
Blinding	20a	Who was blinded after assignment to interventions (eg, participants, care providers, outcome assessors, data analysts)	10
	20b	If blinded, how blinding was achieved and description of the similarity of interventions	NA
Statistical methods	21a	Statistical methods used to compare groups for primary and secondary outcomes, including harms	11-12
	21b	Definition of who is included in each analysis (eg, all randomised participants), and in which group	11-12
	21c	How missing data were handled in the analysis	NA
	21d	Methods for any additional analyses (eg, subgroup and sensitivity analyses), distinguishing prespecified from post hoc	11
**Results**			
Participant flow, including flow diagram	22a	For each group, the numbers of participants who were randomly assigned, received intended intervention, and were analysed for the primary outcome	13-14, Figure 1
	22b	For each group, losses and exclusions after randomisation, together with reasons	13-14, Figure 1
Recruitment	23a	Dates defining the periods of recruitment and follow-up for outcomes of benefits and harms	Detailed in main trial report
	23b	If relevant, why the trial ended or was stopped	NA
Intervention and comparator delivery	24a	Intervention and comparator as they were actually administered (eg, where appropriate, who delivered the intervention/comparator, how participants adhered, whether they were delivered as intended (fidelity))	13
	24b	Concomitant care received during the trial for each group	13
Baseline data	25	A table showing baseline demographic and clinical characteristics for each group	31-32

Numbers analysed, outcomes and estimation	26	For each primary and secondary outcome, by group:<br>● the number of participants included in the analysis<br>● the number of participants with available data at the outcome time point<br>● result for each group, and the estimated effect size and its precision (such as 95% confidence interval)<br>● for binary outcomes, presentation of both absolute and relative effect size	33
Harms	27	All harms or unintended events in each group	14-16
Ancillary analyses	28	Any other analyses performed, including subgroup and sensitivity analyses, distinguishing pre-specified from post hoc	14-16
**Discussion**			
Interpretation	29	Interpretation consistent with results, balancing benefits and harms, and considering other relevant evidence	17-19
Limitations	30	Trial limitations, addressing sources of potential bias, imprecision, generalisability, and, if relevant, multiplicity of analyses	20-21
